# Microwave-Intensified Hofmann Carbylamine Reaction Enabled by Span^®^ 80: Rapid Access to Isocyanides in a Biphasic System

**DOI:** 10.3390/molecules31152620

**Published:** 2026-07-27

**Authors:** Wender Alves Silva, Sayuri Cristina Santos Takada, Saulo Tarso Alves Passos

**Affiliations:** 1 Laboratory for Bioactive Compound Synthesis, Institute of Chemistry, University of Brasilia, Campus Universitario Darcy Ribeiro, Brasilia 70904-970, Brazil; stpassos@gmail.com; 2 Genetic Resources and Biotechnology, Brazilian Agricultural Research Corporation (Embrapa), Brasilia 70770-917, Brazil; sayuri.takada@embrapa.br

**Keywords:** isocyanides, Hofmann carbylamine reaction, microwave-assisted synthesis, biphasic system

## Abstract

Isocyanides constitute a valuable class of organic compounds widely employed in multicomponent reactions (MCRs), heterocycle synthesis, and drug discovery. However, their broader application is limited by low commercial availability and hazardous classical synthetic methods. Herein, we report a greener microwave-assisted protocol for the efficient synthesis of isocyanides through a modified Hofmann carbylamine reaction. The methodology is based on the in situ generation of dichlorocarbene from near-stoichiometric amounts of chloroform and sodium hydroxide, using Span^®^ 80 (sorbitan monooleate) as a biodegradable phase-transfer catalyst under microwave irradiation. Importantly, the protocol eliminates the need for aqueous workup procedures, thereby reducing waste generation and improving the overall sustainability of the process. A broad range of aliphatic-, aromatic-, and amino-acid-derived isocyanides was obtained in moderate-to-excellent isolated yields (43–96%) within 30 min, without isolation of intermediates. Compared with conventional approaches, the proposed methodology avoids toxic dehydrating agents such as POCl_3_ and phosgene, eliminates the need for formamide precursors, and reduces hazardous by-products associated with dehydration processes. The protocol is operationally simple, scalable, and represents a practical, convergent, and environmentally more benign alternative for isocyanide synthesis.

## 1. Introduction

**Figure 1:**
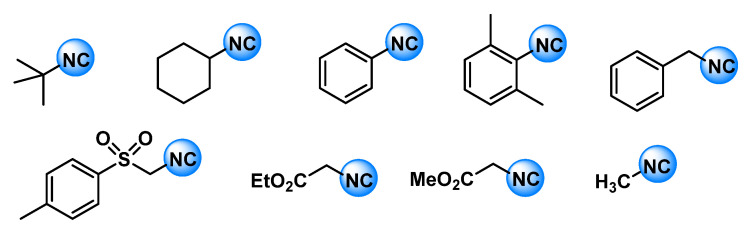
Most common isocyanides commercially available.

Isocyanides (isonitriles or carbylamines) constitute a distinctive class of organic compounds characterized by the terminal R–N^+^≡C^−^ functional group and an unusual electronic structure that enables both nucleophilic and electrophilic reactivity [1]. Owing to these unique properties, isocyanides have become indispensable building blocks in modern organic synthesis, particularly in the construction of heterocyclic frameworks and multicomponent reactions (MCRs) (Figure 1) [2]. A privileged contribution was the development of the Passerini three-component reaction and its subsequent evolution in the Ugi reaction, which significantly expanded the synthetic utility of isocyanides and established them as key intermediates in diversity-oriented synthesis, medicinal chemistry, and drug discovery [3,4,5,6,7,8,9]. In addition to their applications in synthetic chemistry, isocyanides have attracted considerable interest in materials science, supramolecular chemistry, molecular electronics, and organometallic chemistry owing to their isolobal relationship with carbon monoxide and their versatile coordination behavior [10,11,12,13,14].

Beyond their synthetic relevance, naturally occurring isocyanides have been identified in a wide range of terrestrial and marine organisms and display diverse biological activities [15,16,17]. Representative examples include axisonitrile-1, xanthocillin, Kalihinol A, 7,20-diisocyanoadocane, and 9-isocyanopupukeanane [18,19,20,21,22]. These compounds have been associated with antimicrobial, anti-inflammatory, and antimalarial properties, underscoring the ecological and pharmacological significance of the isocyanide functionality [23,24,25,26].

Despite their broad utility, access to isocyanides remains largely dependent on laboratory synthesis. Historically, isocyanides have been prepared through several approaches, including reactions involving silver cyanide, organolithium reagents, and the dehydration of N-formamides [27,28,29,30]. Among these methods, formamide dehydration remains the most widely employed strategy because of its broad substrate scope and reliable performance [31,32,33,34,35]. Nevertheless, these protocols generally require the prior preparation of formamides and often involve multiple synthetic and purification steps. Alternative methodologies have therefore been explored to improve operational efficiency, safety, and sustainability in isocyanide synthesis.

An attractive alternative is the Hofmann carbylamine reaction, first reported in 1867, in which primary amines react with dichlorocarbene generated in situ from chloroform under strongly basic conditions [36]. Unlike formamide-based approaches, the Hofmann reaction provides direct access to isocyanides from primary amines without the need for preformed intermediates. The transformation occurs in a biphasic medium and generally requires phase-transfer catalysis to facilitate hydroxide transport across the aqueous–organic interface [37]. Although the reaction is fairly recognized as a qualitative test for primary amines [38], its potential as a preparative method for isocyanide synthesis remains comparatively underexplored.

**Scheme 1:**
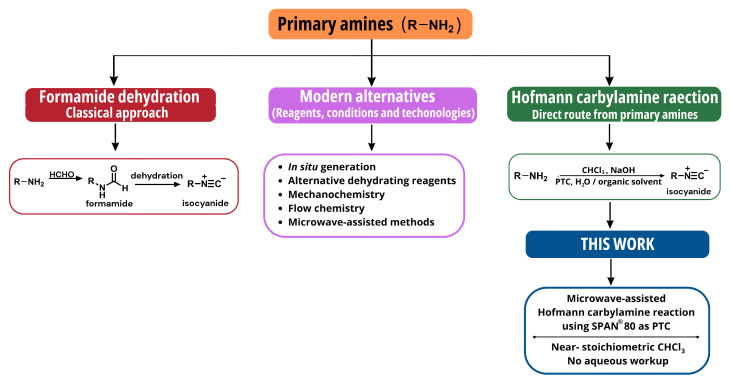
Representative approaches for the synthesis of isocyanides.

Recent efforts to improve isocyanide synthesis have included in situ isocyanide generation strategies, alternative dehydrating reagents, mechanochemical protocols, continuous-flow processes, and microwave-assisted methodologies [39,40,41,42,43,44]. These developments have contributed to improved efficiency and process intensification; however, opportunities are needed to further simplify reaction conditions and reduce reagent consumption. In the specific case of the Hofmann reaction, most reported protocols continue to rely on large excesses of chloroform, conventional quaternary ammonium phase-transfer catalysts, or prolonged heating periods, as shown in Scheme 1 [44,45].

**Figure 2:**
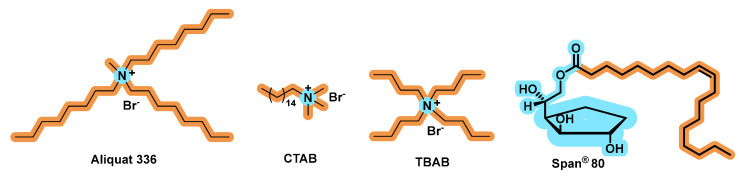
Chemical structures of the phase−transfer catalysts evaluated in this study. The hydrophilic headgroup is highlighted in light blue and the hydrophobic moiety in orange.

Phase-transfer catalysis plays a central role in the Hofmann carbylamine reaction because efficient integration between the aqueous and organic phases is essential for dichlorocarbene generation. Conventional protocols typically employ quaternary ammonium salts such as Aliquat 336, cetyltrimethylammonium bromide (CTAB) and tetrabutylammonium bromide (TBAB) [45,46]. The fundamental concepts and applications of phase-transfer catalysis have been extensively discussed by Starks, Liotta, and Halpern, highlighting the importance of catalyst structure in controlling interfacial reactivity [47]. In contrast, Span^®^ 80 (sorbitan monooleate) is a commercially available nonionic surfactant widely used in emulsion and interfacial systems, as shown in Figure 2. Its amphiphilic structure, commercial availability, and favorable environmental profile make it an attractive candidate for promoting mass transfer in biphasic reaction media. 

As part of our continuing interest in microwave-assisted organic synthesis [48], we sought to explore the combination of microwave irradiation and biodegradable surfactant-mediated phase-transfer catalysis for the Hofmann carbylamine reaction. Inspired by recent advances aimed at simplifying isocyanide synthesis and purification workflows [49], we investigated the use of Span^®^ 80 as a phase-transfer promoter in a microwave-assisted biphasic system.

Herein, we report a microwave-assisted Hofmann carbylamine protocol employing near-stoichiometric quantities of chloroform and Span^®^ 80 as a biodegradable phase-transfer catalyst. The methodology enables the rapid synthesis of a variety of aliphatic, aromatic, heteroaromatic, and amino acid methyl ester derivatives under operationally simple conditions. An additional feature of the protocol is the elimination of conventional aqueous workup procedures, thereby reducing solvent consumption and simplifying product isolation. To the best of our knowledge, the use of Span^®^ 80 as a phase-transfer promoter in a microwave-assisted Hofmann carbylamine reaction has not been previously reported [50]. Importantly, the term “greener” is used herein in a comparative sense, reflecting improvements relative to conventional methodologies rather than implying absolute sustainability. Within this context, the present strategy provides a practical and operationally efficient approach to isocyanide synthesis while reducing reagent consumption, waste generation, and overall process complexity.

## 2. Results and Discussion

Previous microwave-assisted Hofmann protocols typically rely on conventional phase-transfer catalysts and often require extended reaction times or excess chloroform [44]. In contrast, the present methodology integrates microwave irradiation with a biodegradable surfactant-mediated phase-transfer system, enabling efficient carbene generation and capture within significantly shorter reaction times.

To establish optimal conditions for the modified Hofmann isocyanide synthesis, an initial optimization study was performed using benzylamine as the model substrate. Key reaction parameters—including the amine-to-chloroform ratio, base loading, temperature, reaction time, and the nature of the phase-transfer catalyst, Span^®^ 80—were systematically examined based on the classical methodology described by Gokel and co-workers [37]. The results of this screening are summarized in Table 1.

Although the boiling point of chloroform is approximately 61 °C under atmospheric pressure, reactions performed in sealed microwave vessels develop autogenous pressure, allowing the solvent to remain in the liquid phase at substantially higher temperatures. This feature enables efficient carbene generation while using only near-stoichiometric amounts of chloroform.

**Scheme 2:**
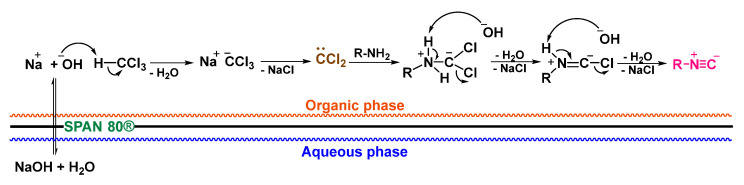
Proposed phase-transfer-catalyzed mechanism for isocyanide synthesis.

The optimized protocol provides an operationally practical approach for the preparation of representative known isocyanides from different classes of primary amines under microwave-assisted conditions. The methodology affords the desired products within short reaction times while using reduced amounts of chloroform and a biodegradable phase-transfer catalyst. The requirement for excess sodium hydroxide is consistent with the mechanistic demands of the Hofmann carbylamine reaction (Scheme 2). As established in prior studies, one equivalent of hydroxide promotes chloroform dehydrohalogenation to generate dichlorocarbene, while two additional equivalents are necessary to neutralize the hydrogen chloride formed during conversion of the amine–carbene intermediate to the corresponding isocyanide [37]. Insufficient base results in residual acidity in the reaction medium, which promotes α-addition to the nascent isocyanide and leads to a significant decrease in yield. Notably, although sodium hydroxide is employed in excess, its use generates only benign inorganic byproducts (NaCl and water). In this context, the present methodology represents a substantially safer alternative to traditional dehydration protocols that rely on highly hazardous chlorinated reagents, such as POCl_3_ or phosgene.

Although dichlorocarbene is intrinsically reactive, it is generated in situ and rapidly intercepted by the amine substrate, thereby minimizing exposure risks and avoiding the handling of isolable toxic intermediates. A slight excess of amine further improves reaction efficiency by ensuring prompt carbene capture and suppressing competing pathways such as insertion, hydrolysis, or thermal decomposition. In addition, the amine-rich environment helps buffer transient acidity and compensates for phase-distribution limitations inherent to the biphasic system, while also mitigating evaporative losses in the case of volatile substrates. Collectively, these factors contribute to the high yields observed under the optimized conditions (Table 1).

Following establishment of the optimal stoichiometry and temperature profile, we evaluated the influence of surfactant structure on reaction performance (Table 2). Comparative analysis revealed that the nonionic, biodegradable surfactant Span^®^ 80 outperformed conventional quaternary ammonium phase-transfer catalysts, affording higher conversions and cleaner reaction profiles. The superior performance observed for Span^®^ 80 highlights the potential of biodegradable nonionic surfactants as effective phase-transfer promoters in strongly biphasic systems. Whereas traditional Hofmann protocols typically rely on quaternary ammonium salts, the present results indicate that sorbitan-derived surfactants can deliver comparable—or even improved—catalytic efficiency while providing a more environmentally acceptable alternative. The amphiphilic architecture of Span^®^ 80, combined with the concentrated biphasic reaction medium, likely enhances phase-transfer efficiency by facilitating hydroxide transport across the aqueous–organic interface. This improved interfacial communication accelerates the in situ generation of dichlorocarbene and promotes its efficient trapping by the amine substrate, thereby minimizing diffusional limitations commonly associated with Hofmann-type systems.

Compared with methodologies reported under conventional heating conditions, microwave irradiation significantly intensified the process, reducing the reaction time from approximately three hours under reflux to only 30 min at 80 °C without compromising the yield [34,37]. This combination of microwave activation and surfactant-assisted phase-transfer catalysis promotes effective reaction intensification by enhancing interfacial mass transfer and facilitating rapid in situ generation and capture of dichlorocarbene. Under the optimized conditions (1.2:1 amine-to-chloroform ratio and 4.0 equivalents of NaOH), benzyl isocyanide was obtained in 95% isolated yield. The methodology also proved readily scalable: increasing the reaction scale from 1 mmol to 10 mmol proceeded without operational difficulties or loss of efficiency, highlighting the robustness and preparative utility of the microwave-assisted Hofmann—Span^®^ 80 protocol. Importantly, Span^®^ 80 remained stable under the optimized reaction conditions, despite the presence of an ester functionality within its structure. To further assess its stability, a simple and direct control experiment was performed under the optimized conditions in the absence of the amine substrate. Subsequent NMR analysis of the recovered surfactant revealed no detectable decomposition over the course of the reaction, thereby supporting the operational stability of Span^®^ 80 under the employed conditions.

To systematically evaluate the practical advantages associated with the elimination of conventional aqueous workup procedures, a comparative experiment was conducted using benzyl isocyanide as a model substrate (Table 3). Direct purification of the crude reaction mixture without aqueous extraction resulted in improved isolated yields, reduced solvent consumption, and a simplified operational workflow compared with the conventional extraction-based protocol. These results further support the operational and environmental benefits of the present methodology.

**Scheme 3:**
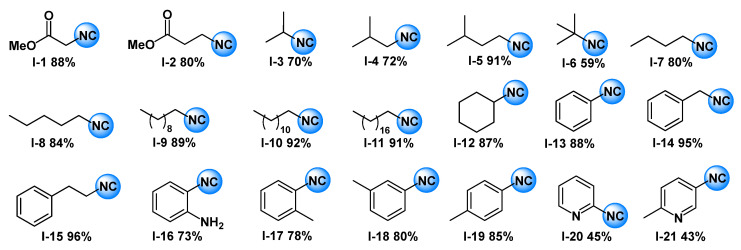
Isocyanides synthesized and their respective yields. % yields shown in this scheme are the isolated yields of isocyanides.

To assess the scope and robustness of the methodology, a representative set of primary amines was subjected to the optimized conditions. Isocyanides I-1 to I-15 were synthesized on a 10 mmol scale, demonstrating the preparative applicability of the protocol. In contrast, isocyanides I-16 to I-21 were obtained on a 1.0 mmol scale solely due to the limited availability of the corresponding starting materials in the laboratory (Scheme 3).

The conversion of amino acid methyl ester derivative substrates proceeded efficiently (>80% yield), demonstrating the compatibility of the methodology with ester-containing, base-sensitive functional groups. Under conventional Hofmann carbylamine conditions, such functionalities are often susceptible to base-promoted hydrolysis because of the large excesses of concentrated sodium hydroxide typically employed. In contrast, the biphasic reaction system used herein, combined with a reduced amount of aqueous base, enabled efficient isocyanide formation without detectable ester hydrolysis. This enhanced chemoselectivity is likely attributable to the rapid formation of the isocyanide and its preferential partitioning into the organic phase, thereby minimizing its exposure to the aqueous alkaline medium. Aliphatic amines such as decylamine, dodecylamine, and octadecylamine furnished stable isocyanides in yields above 85%. Lower yields occasionally observed for low-molecular-weight aliphatic isocyanides (linear or branched) were attributed to their high volatility, which leads to material loss during solvent removal following chromatographic purification. Cyclohexylamine also underwent smooth conversion under the optimized conditions. Aromatic amines—including benzylamine derivatives, aniline, and *o*-, *m*-, and *p*-toluidine—provided the corresponding isocyanides in moderate-to-excellent yields (73–85%), demonstrating that the optimized reaction conditions are compatible with the representative substrates examined in this work. Heterocyclic isocyanides with the isocyano group positioned ortho to a heteroatom are known to be relatively unstable and prone to polymerization. Pyridine-derived isocyanides, in particular, are frequently described as highly unstable compounds that must be used immediately after isolation. Both 2-isocyanopyridine (I-20, 45%) and 4-(isocyanomethyl)pyridine (I-21, 43%) were obtained, although in modest yields due to the aforementioned stability issues. Despite being stable enough for isolation and NMR characterization, these heterocyclic isocyanides gradually decompose at room temperature and cannot be stored long term.

To compare the performance of our methodology with that of traditional protocols, we conducted a comparative analysis of key green chemistry parameters, with particular emphasis on the environmental factor (E-factor), a widely accepted metric in which lower values indicate superior overall process performance [51]. Compound I-15 was compared with two additional methodologies for isocyanide synthesis via POCl_3_-mediated dehydration of formamides (Ugi reaction) [52,53,54]. Detailed calculations are provided in the Supplementary Materials; nevertheless, the advantages of our protocol are readily apparent. 

As expected, conventional routes exhibit substantially higher E-factors due to the excessive use of solvents during aqueous workup procedures and the intrinsically diluted nature of these reactions. An important aspect that should be taken into consideration is that none of these studies includes the preparation of the corresponding formamides or their environmental impact on isocyanide production in the calculations, thereby rendering the overall assessment overly simplified. In contrast, our microwave-assisted Hofmann–Span^®^ 80 process operates at higher reaction concentrations and completely eliminates the aqueous workup step, resulting in significantly reduced waste generation. These improvements translate into markedly lower E-factors, the formation of non-toxic residues, and enhanced energy efficiency, thereby reinforcing the sustainability advantages of the methodology and confirming that our protocol constitutes an operationally simpler and less waste-generating alternative compared with traditional syntheses of isocyanides. 

In Table 4, we summarize all previously reported E-factor values for the synthesis of phenethyl isocyanides using POCl_3_-mediated methodologies.

Overall, the combination of microwave irradiation, near-stoichiometric chloroform, and the environmentally acceptable surfactant Span^®^ 80 provides a greener variant of the Hofmann reaction. The method eliminates the need for dehydrating agents such as POCl_3_ and phosgene and avoids excessive solvent waste, while maintaining scalability and reproducibility. These findings reinforce the potential of this protocol as a sustainable platform for isocyanide synthesis, suitable for both instructional and preparative applications in modern organic chemistry.

## 3. Materials and Methods

All reagents and solvents were purchased from Sigma-Aldrich–Merck (St. Louis, MO, USA). Microwave-assisted reactions were performed using a Biotage Initiator+ instrument (Biotage, Uppsala, Sweden) in sealed borosilicate reaction vials. Reactions were conducted under autogenous pressure with dynamic microwave power control, continuous temperature monitoring via an internal fiber-optic probe, simultaneous air cooling, and magnetic stirring. NMR spectra were recorded at 25 °C on a Bruker Avance 600 spectrometer (Bruker BioSpin, Billerica, MA, USA; 600 MHz for ^1^H and 151 MHz for ^13^C) and on a Varian Mercury Plus spectrometer (Varian, Inc., Palo Alto, CA, USA; 300 MHz for ^1^H and 75 MHz for ^13^C), with TMS as the internal standard and CDCl_3_ as the solvent. Reaction progress was monitored by thin-layer chromatography (TLC) on precoated 0.25 mm Kieselgel 60 F_254_ plates (Merck KGaA, Burlington, MA, USA). Visualization was achieved under UV light (254 nm) or by spraying a 10% phosphomolybdic acid solution in ethanol, followed by heating at 200 °C until blue spots appeared.

Use of AI-Assisted Tools: AI-assisted tools (GPT-5.5, OpenAI) were used solely for English grammar correction and language editing. All scientific content, data interpretation, and conclusions were developed, verified, and approved exclusively by the authors. 

Although the reaction minimizes the use of toxic and corrosive reagents, chloroform and isocyanides must be handled in a well-ventilated fume hood due to the strong odor. All waste solutions were neutralized prior to disposal according to institutional safety protocols.

### 3.1. General Microwave-Assisted Synthesis of Isocyanides on a 1.0 mmol Scale

A mixture of the primary amine (1.2 mmol) and chloroform (1.0 mmol) was combined with sodium hydroxide (4.0 mmol and 0.3 mL of distilled water) and Span^®^ 80 (0.02 mmol) as a biodegradable phase-transfer catalyst, in a 5 mL sealed glass tube. The biphasic mixture was stirred magnetically and heated under microwave irradiation at 80 °C for 30 min. After cooling to room temperature, the reaction mixture was adsorbed onto silica gel (10 g, 230–400 mesh) and directly loaded onto a prepacked silica column (230–400 mesh). Purification using a heptane/2-methyltetrahydrofuran (8:2) eluent afforded the corresponding isocyanide in 43–86% yield. Products were characterized by NMR data, which were consistent with the expected structures.

### 3.2. General Microwave-Assisted Synthesis of Isocyanides on a 10 mmol Scale

In a 20 mL sealed glass tube, the corresponding primary amine (12 mmol) and chloroform (10 mmol) were combined with sodium hydroxide (40 mmol, 3 mL of distilled water) and Span^®^ 80 (0.2 mmol) as a biodegradable phase-transfer catalyst. The biphasic mixture was stirred magnetically and heated under microwave irradiation at 80 °C for 30 min. After cooling to room temperature, the reaction mixture was adsorbed onto silica gel (20 g, 230–400 mesh) and directly loaded onto a prepacked silica column (230–400 mesh). Purification using a heptane/2-methyltetrahydrofuran (8:2) eluent afforded the corresponding isocyanide in 59–96% yield. Products were characterized by NMR data, consistent with the expected structures.

**(I-1) Methyl isocyanoacetate.** [55] ^1^H NMR (600 MHz, CDCl_3_) δ: 4.26 (s, 2H), 3.84 (s, 3H); ^13^C NMR (151 MHz, CDCl_3_) δ: 164.4, 161.4, 53.4, 43.4 (t, *J* = 6.2 Hz). Yield 88%; 872.0 mg. HRESI-MS (ESI-QTOF) *m*/*z* calculated for C_4_H_6_NO_2_^+^ [M + H]^+^: 100.0393, found [M + H]^+^: 100.0393. Purified product: colorless liquid.
**(I-2) Methyl 3-isocyanopropanoate.** [56] ^1^H NMR (600 MHz, CDCl_3_) δ: 3.72–3.71 (m, 3H), 3.68–3.65 (m, 2H), 2.73–2.69 (m, 2H); ^13^C NMR (151 MHz, CDCl_3_) δ: 169.7, 157.2 (t, *J* = 4.2 Hz), 52.1, 37.0 (t, *J* = 7.3 Hz), 33.8. Yield 80%; 905.0 mg. HRMS (ESI-QTOF) *m*/*z* calculated for C_5_H_8_NO_2_^+^ [M + H]^+^: 114.0550, found [M + H]^+^: 114.0551. Purified product: colorless liquid.
**(I-3) Isopropyl isocyanide.** [55] ^1^H NMR (600 MHz, CDCl_3_): δ 3.79–3.74 (m, 1H), 1.38–1.37 (s, 6H); ^13^C NMR (151 MHz, CDCl_3_): δ 153.6, 45.7, 23.4. Yield 70%; 483.7 mg. HRESI-MS (ESI-QTOF) *m*/*z* calculated for C_4_H_10_NO^+^ [M + H + H_2_O]^+^: 88.0757, found [M + H + H_2_O]^+^: 88.0755. Purified product: colorless liquid.
**(I-4) Isobutyl isocyanide**. [55] ^1^H NMR (600 MHz, CDCl_3_) δ: 3.38–3.35 (m, 2H), 1.78–1.72 (m, 1H), 0.91 (d, *J* = 6.6 Hz, 6H); ^13^C NMR (151 MHz, CDCl_3_) δ: 155.8 (t, *J* = 4.4 Hz), 46.1, 24.9, 21.7. Yield 72%; 598.6 mg. HRESI-MS (ESI-QTOF) *m*/*z* calculated for C_5_H_12_NO^+^ [M + H + H_2_O]^+^: 102.0913, found [M + H + H_2_O]^+^: 102.0912. Purified product: colorless liquid.
**(I-5) 1-Isocyano-3-methylbutane**. [57] ^1^H NMR (600 MHz, CDCl_3_) δ: 3.38–3.35 (m, 2H), 1.78-1.72 (m, 1 H), 1.55–1.52 (m, 2H), 0.91 (m, 6H); ^13^C NMR (151 MHz, CDCl_3_) δ: 157.5 (t, *J* = 5.8 Hz), 34.9 (t, *J* = 6.4 Hz), 27.6, 21.7. Yield 91%; 884.2 mg. HRESI-MS (ESI-QTOF) *m*/*z* calculated for C_6_H_14_NO^+^ [M + H + H_2_O]^+^: 116.1075, found [M + H + H_2_O]^+^: 116.1073. Purified product: colorless liquid.
**(I-6) tert-Butyl isocyanide.** [55] ^1^H NMR (600 MHz, CDCl_3_) δ: 1.46 [s, 9H]; ^13^C NMR (151 MHz, CDCl_3_): δ 152.4; 30.5. Yield 59%; 490.5 mg. HRESI-MS (ESI-QTOF) *m*/*z* calculated for C_5_H_12_NO^+^ [M + H + H_2_O]^+^: 102.0913, found [M + H + H_2_O]^+^: 102.0911. Purified product: colorless liquid.
**(I-7) Butyl isocyanide.** [55] ^1^H NMR (600 MHz, CDCl_3_) δ: 3.40–3.37 [m, 2H], 1.68–1.64 [m, 2H], 1.50–1.45 [m, 2H], 0.95 [t, 3H, *J* = 7.3 Hz]; ^13^C NMR (151 MHz, CDCl_3_) δ: 155.5, 41.2 (t, *J* = 6.5 Hz), 30.8, 19.5, 13.1. Yield 80%; 665.1 mg. HRESI-MS (ESI-QTOF) *m*/*z* calculated for C_5_H_12_NO^+^ [M + H + H_2_O]^+^: 102.0913, found [M + H + H_2_O]^+^: 102.0912. Purified product: colorless liquid.
**(I-8) Amyl isocyanide.** [55] ^1^H NMR (600 MHz, CDCl_3_) δ 3.31–3.28 (m, 2H), 1.60–1.54 (m, 2H), 1.41–1.35 (m, 2H), 0.86 (t, *J* = 7.4 Hz, 3H); ^13^C NMR (151 MHz, CDCl_3_) δ 155.7, 41.2 (t, *J* = 6.4 Hz), 30.9, 21.4, 19.4, 12.5. Yield 84%, 816.2 mg. HRESI-MS (ESI-QTOF) *m*/*z* calculated for C_6_H_14_NO^+^ [M + H + H_2_O]^+^: 116.1070, found [M + H + H_2_O]^+^: 116.1071. Purified product: colorless liquid.
**(I-9) 1-Isocyanodecane.** [55] ^1^H NMR (600 MHz, CDCl_3_) δ: 3.34 (t, *J* = 6.8 Hz, 2H), 1.66–1.63 (m, 2H), 1.41–1.38 (m, 2H), 1.27–1.24 (m, 12H), 0.86–0.84 (m, 3H); ^13^C NMR (151 MHz, CDCl_3_) δ: 155.5, 41.4 (t, *J* = 6.3 Hz), 31.7, 29.4, 29.3, 29.2, 29.1, 28.9, 28.5, 26.1, 13.9. Yield 89%; 1489.0 mg. HRESI-MS (ESI-QTOF) *m*/*z* calculated for C_11_H_24_NO^+^ [M + H + H_2_O]^+^: 186.1852, found [M + H + H_2_O]^+^: 186.1851. Purified product: pale yellow pasty material.
**(I-10) Dodecyl isocyanide.** [55] ^1^H NMR (600 MHz, CDCl_3_) δ: 3.40–3.38 [m, 2H], 1.72–1.66 [m, 2H], 1.47–1.42 [m, 2H], 1.34–1.26 [m, 16H], 0.91–0.89 [m, 3H]; NMR ^13^C (151 MHz, CDCl_3_) δ: 155.6, 41.6, 31.9, 29.6, 29.5, 29.4, 29.3, 29.1, 28.6, 26.3, 22.8, 14.1. Yield 92%; 1797.2 mg. HRESI-MS (ESI-QTOF) *m*/*z* calculated for C_13_H_28_NO^+^ [M + H + H_2_O]^+^: 214.2165, found [M + H + H_2_O]^+^: 214.2163. Purified product: pale yellow pasty material.
**(I-11) Octadecyl isocyanide.** [55] ^1^H NMR (600 MHz, CDCl_3_) δ: 3.41–3.38 [m, 2H], 1.71–1.68 [m, 2H], 1.48–1.43 [m, 2H], 1.35–1.28 [m, 28H], 0.91–0.89 [m, 3H]; NMR ^13^C (151 MHz, CDCl_3_) δ: 155.6, 41.6, 31.9, 29.7, 29.6, 29.5, 29.4, 29.1, 26.3, 22.8, 14.1. Yield 91%; 2543.6 mg. HRESI-MS (ESI-QTOF) *m*/*z* calculated for C_19_H_40_NO^+^ [M + H + H_2_O]^+^: 298.3104, found [M + H + H_2_O]^+^: 298.3104. Purified product: light-brown paste.
**(I-12) Cyclohexyl isocyanide.** [55] ^1^H NMR (600 MHz, CDCl_3_) δ: 3.63–3.59 [m, 1H], 1.91–1.88 [m, 2H], 1.79–1.66 [m, 4H], 1.51–1.35 [m, 4H]; ^13^C NMR (151 MHz, CDCl_3_) δ: 153.9, 51.7, 32.7, 24.9, 22.7. Yield 87%; 949.8 mg. HRESI-MS (ESI-QTOF) *m*/*z* calculated for C_7_H_14_NO^+^ [M + H + H_2_O]^+^: 128.1070, found [M + H + H_2_O]^+^: 128.1069. Purified product: colorless liquid.
**(I-13) Phenyl isocyanide.** [55] NMR ^1^H (600 MHz, CDCl_3_) δ: 7.44–7.40 (m, 5H); ^13^C NMR (151 MHz, CDCl_3_) δ: 163.8, 146.3, 129.3, 129.1, 126.3. Yield 88%; 907.5 mg. HRESI-MS (ESI-QTOF) *m*/*z* calculated for C_7_H_6_N^+^ [M + H]^+^: 104.0495, found [M + H]^+^: 104.0495. Purified product: pale yellow liquid.
**(I-14) (Isocyanomethyl)benzene.** [58] ^1^H NMR (600 MHz, CDCl_3_) δ: 7.42–7.40 (m, 2H), 7.38–7.35 (m, 3H), 4.64 (s, 2H); ^13^C NMR (151 MHz, CDCl_3_) δ: 157.4 (t, *J* = 5.3 Hz), 132.1, 128.7, 128.1, 126.6, 45.2 (t, *J* = 7.3 Hz). Yield 95%; 1112.1 mg. HRESI-MS (ESI-QTOF) *m*/*z* calculated for C_7_H_7_^+^ [M + H−HCN]^+^: 91.0542, found [M+H-HCN]^+^: 91.0542. Purified product: colorless liquid.
**(I-15) 2-(Isocyanoethyl)benzene.** [55] ^1^H NMR (600 MHz, CDCl_3_)) δ 7.43–7.22 (m, 5H), 3.61 (tt, *J* = 7.1, 1.9 Hz, 2H), 3.00 (tt, *J* = 7.1, 2.2 Hz, 2H); ^13^C NMR (151 MHz, CDCl_3_) δ 156.5 (t, *J* = 5.6 Hz), 136.6 (br t), 128.7, 128.7, 127.2, 42.9 (t, *J* = 6.6 Hz), 35.5 Yield 96%; 1259.3 mg. HRMS (ESI) *m*/*z* calculated for C_8_H_9_^+^ [M + H–HCN]+: 105.0699; found, 105.0698. Purified product: pale yellow liquid.
**(I-16) 2-Isocyanoaniline**. [55] ^1^H NMR (600 MHz, CDCl_3_) δ: 7.24–7.18 (m, 2H), 6.80–6.71 (m, 2H), 4.19 (bs, 2H); ^13^C NMR (151 MHz, CDCl_3_) δ: 167.1, 142.7, 130.3, 126.7, 118.1, 115.5. Yield 73%; 86.24 mg. HRESI-MS (ESI-QTOF) *m*/*z* calculated for C_7_H_7_N_2_^+^ [M+H]^+^: 119.0604, found [M + H]^+^: 119.0602. Purified product: pale yellow liquid.
**(I-17) 1-Isocyano-2-methylbenzene.** [59] ^1^H NMR (600 MHz, CDCl_3_) δ: 7.35–7.33 (m, 1H), 7.30–7.28 (m, 2H), 7.22–7.20 (m, 1H), 2.43 (s, 3H); ^13^C NMR (151 MHz, CDCl_3_) δ: 165.5 (t, *J* = 5.8 Hz), 134.9, 130.5, 129.2, 126.6, 126.5, 18.5. Yield 78%; 91.38 mg. HRESI-MS (ESI-QTOF) *m*/*z* calculated for C_8_H_8_N^+^ [M + H]^+^: 118.0651, found [M + H]^+^: 118.0652. Purified product: colorless liquid.
**(I-18) 1-Isocyano-3-methylbenzene.** [59] ^1^H NMR (600 MHz, CDCl_3_) δ: 7.25–7.23 (m, 1H), 7.18–7.14 (m, 3H), 2.36 (s, 3H); ^13^C NMR (151 MHz, CDCl_3_) δ: 163.3 (t, *J* = 5.6 Hz), 139.6, 130.1, 129.1, 126.8, 123.4, 21.1. Yield 80%; 93.72 mg. HRESI-MS (ESI-QTOF) *m*/*z* calculated for C_8_H_8_N^+^ [M+H]^+^: 118.0651, found [M+H]^+^: 118.0652. Purified product: colorless liquid.
**(I-19) 1-Isocyano-4-methylbenzene.** [59] ^1^H NMR (600 MHz, CDCl_3_) δ: 7.26 (d, *J* = 8.2 Hz, 2H), 7.18 (d, *J* = 8.2 Hz, 2H), 2.37 (s, 3H); ^13^C NMR (151 MHz, CDCl_3_) δ: 163.1 (t, *J* = 5.6 Hz), 139.7, 129.9, 126.1, 124.0 (t, *J* = 13.8 Hz), 21.3. Yield 85%; 99.58 mg. HRESI-MS (ESI-QTOF) *m*/*z* calculated for C_8_H_8_N^+^ [M + H]^+^: 118.0651, found [M + H]+: 118.0652. Purified product: colorless liquid.
**(I-20) 2-Isocyanopyridine.** [58] ^1^H NMR (600 MHz, CDCl_3_) δ: 8.50 (dd, *J* = 4.9, 1.1 Hz, 1H), 7.82 (td, *J* = 7.8, 1.9 Hz, 1H), 7.40–7.35 (m, 2H); ^13^C NMR (151 MHz, CDCl_3_) δ: 163.8, 149.8, 140.4 (t, *J* = 17.2 Hz), 138.7, 124.7, 121.4. Yield 45%; 53.16 mg. HRESI-MS (ESI-QTOF) *m*/*z* calculated for C_6_H_7_N_2_O^+^ [M + H + H_2_O]^+^: 123.0558, found [M + H + H_2_O]^+^: 123.0560. Purified product: pale yellow liquid.
**(I-21) 5-Isocyano-2-methylpyridine**. [55] ^1^H NMR (600 MHz, CDCl_3_) δ: 8.56 (d, *J* = 2.4 Hz, 1H), 7.57 (dd, *J* = 8.3, 2.4 Hz, 1H), 7.21 (d, *J* = 8.3 Hz, 1H), 2.59 (s, 3H); ^13^C NMR (151 MHz, CDCl_3_) δ: 168.0, 159.5, 146.7, 133.5, 123.4, 24.4. Yield 43%, 44.77 mg. HRESI-MS (ESI-QTOF) *m*/*z* calculated for C_7_H_9_N_2_O^+^ [M + H + H_2_O]^+^: 137.0715, found [M + H + H_2_O]^+^: 137.0716. Purified product: pale yellow liquid.

## 4. Conclusions

This work demonstrates that the microwave-assisted Hofmann–Span^®^ 80 protocol transforms the classical Hofmann carbylamine reaction into a more operationally practical and environmentally improved methodology for isocyanide synthesis without compromising substrate applicability. The combination of microwave irradiation, a biodegradable nonionic phase-transfer catalyst, a biphasic reaction medium, and near-stoichiometric amounts of chloroform enables the efficient synthesis of structurally diverse isocyanides in only 30 min under mild reaction conditions. Notably, the successful conversion of ester-containing substrates highlights the improved functional-group compatibility achieved under the optimized conditions. In addition, the elimination of aqueous workup, reduced solvent consumption, lower waste generation (E-factor = 17.20), and the replacement of classical dehydrating agents with a more sustainable protocol further demonstrate the operational, safety, and environmental advantages of the methodology. Importantly, the optimized protocol enables the preparation of a variety of known isocyanides under microwave-assisted biphasic conditions, including representative methyl ester-containing and heterocyclic substrates examined in this work. While microwave reactors may present limitations for large-scale industrial implementation, they are widely accepted as effective tools for reaction intensification at the laboratory and pilot scale. Moreover, microwave irradiation serves as an effective tool for reaction intensification; however, the fundamental chemistry is not restricted to this activation mode and could potentially be implemented under alternative heating regimes, including continuous-flow platforms. Overall, the methodology should be regarded as a greener and safer alternative within the context of established isocyanide synthesis routes. Although the method still employs chloroform—a solvent with inherent toxicity—its use of near-stoichiometric quantities, combined with the elimination of even more hazardous dehydrating agents (POCl_3_ and phosgene), reduced reaction times, and a significant decrease in overall waste generation, collectively represent a meaningful improvement in sustainability. Future work should focus on exploring alternative, greener carbene precursors to further enhance the environmental profile of this transformation.

## Figures and Tables

**Table 1:** Optimization of the microwave-assisted Hofmann carbylamine reaction using benzylamine as model substrate under direct purification conditions.

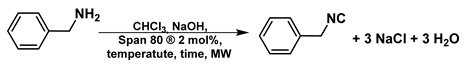					
Entry	Amine:CHCl_3_ (Equiv)	NaOH (Equiv)	Temp (°C)	Time (min)	Yield (%) ^a^
1	1:1	4.0	100	60	70
2	1:1	4.0	100	30	73
3	1:1	4.0	80	60	79
4	1:1	4.0	80	30	82
5	1.1:1	4.0	80	30	85
6	1.2:1	4.0	80	30	95
7	1.3:1	4.0	80	30	89
8	1:1.1	4.0	80	30	85
9	1:1.2	4.0	80	30	90
10	1:1.3	4.0	80	30	92

^a^ All yields correspond to isolated products obtained through direct chromatographic purification without aqueous workup.

**Table 2:** Optimization of the Hofmann carbylamine reaction using benzylamine as model substrate.

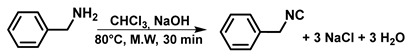		
Entry	Surfactant	Yield (%) ^a^
1	CTAB	68
2	TBAB	75
3	Aliquat 336	86
4	Span^®^ 80	95
5	-	5

^a^ All yields correspond to isolated products obtained through direct chromatographic purification without aqueous workup.

**Table 3:** Comparison between conventional aqueous workup and direct purification protocol.

Entry	Purification Strategy	Yield (%)	Solvent Consumption	Waste Generation
1	Aqueous extraction + chromatography	89	Higher	Higher
2	Direct chromatography (this work)	95	Lower	Lower

**Table 4:** Comparative E-factor analysis of representative isocyanide syntheses.

Process	Yield (%)	E-Factor
Porcal and co-workers [52]	97	63.39 ^a^
Pirali and co-workers [53]	77	83.44 ^a^
Goldeman and co-workers [54]	45	60.19 ^a^
Present study	96	17.20

^a^ Values were calculated excluding the purification step.

## Data Availability

Data are contained within the article and Supplementary Materials.

## Supplementary Materials

The following supporting information can be downloaded at https://www.mdpi.com/article/10.3390/molecules31152620/s1. Detailed ^1^H NMR and ^13^C NMR spectral data for all compounds Figures S1–S21.

## Abbreviations

The following abbreviations are used in this manuscript:

^1^HNMR	proton nuclear magnetic resonance
^13^CNMR	carbon-13 nuclear magnetic resonance
HRESI-MS	high-resolution electrospray ionization mass spectrometry
*p*-TsCl	*p*-toluenesulfonyl chloride
CTAB	cetyltrimethylammonium bromide
TBAB	tetrabutylammonium bromide
Aliquat 336	methyltrioctylammonium chloride
PTC	phase-transfer catalyst
