# Supplementary material for: Microwave-Intensified Hofmann Carbylamine Reaction Enabled by Span® 80: Rapid Access to Isocyanides in a Biphasic System
Source: Molecules. 2026 Jul 27;31(15):2620. doi: 10.3390/molecules31152620 (PMC13467631; doi:10.3390/molecules31152620)
Supplement: Supplementary file 1 [file molecules-31-02620-s001.zip › molecules-4369389-supplementary.pdf]

Supporting Information

Microwave-Intensified Hofmann Carbylamine Reaction Enabled by Span® 80: Rapid Access to Isocyanides in a Biphasic System

Wender Alves Silva, Sayuri Cristina Santos Takada, Saulo Tarso Alves Passos

Contents

General Information S2

General Microwave-Assisted Synthesis of isocyanides S2

Analytical Data S3

Calculation of Green Chemistry Metrics S5

Spectra NMR S10

General Information

All reagents and solvents were purchased from Sigma-Aldrich-Merck (St. Louis, MO, USA). The solvents were from commercial sources and when required were treated and dried as according to the literature. Purification of the products was performed by flash chromatographic column, using silica gel 60, 230-400 mesh ASTM Merck. The reactions were monitored by thin-layer chromatography (TLC) on precoated 0.25mm thick plates of Kieselgel 60 F254 (Burlington, MA, USA), visualization was accomplished by UV light (254 nm) or by spraying a solution of 10% solution of phosphomolybdic acid in ethanol, followed by heating at 200°C for a sufficient duration until blue spots become visible. Microwave-assisted reactions were performed using a Biotage Initiator+ instrument (Uppsala, Sweden) in sealed borosilicate reaction vials. Reactions were conducted under autogenous pressure with dynamic microwave power control, continuous temperature monitoring via an internal fiber-optic probe, simultaneous air cooling, and magnetic stirring. The NMR spectra were recorded at 25 °C on a Bruker Avance 600 spectrometer (Billerica, MA, USA, 600 MHz for 1H and 151 MHz for 13C) and Varian Mercury Plus spectrometer (Palo Alto, CA, USA, 300 MHz for ^{1}H and 75 MHz for ^{13}C) with TMS as an internal standard for deuterated chloroform ($CDCl_3$) as solvent and chemical shifts (δ) are expressed in ppm referenced by the residual solvent signal and the coupling constants (J) in Hertz (Hz). To indicate the multiplicity of the signals the following abbreviations were used: s (singlet), d (doublet), t (triplet), q (quadruplet), m (multiplet), dd (doublet of doublets), ddd (doublet of doublet of doublets), ddt (doublet of doublet of triplets), dt (doublet of triplets), td (triplet of doublets) and tt (triplet of triplets). HRESI-MS (ESI-QTOF) experiments were performed on a Triple Tof 5600 Sciex (Framingham, MA, USA), by flow injection analysis using an Eksigent UltraLC 100 Sciex chromatograph set to a flow rate of 0.3 mL/min.

Safety considerations

Although the reaction minimizes the use of toxic and corrosive reagents, all reactants should be handled in a well-ventilated fume hood due to their potential toxicity and strong odor. All waste solutions were properly neutralized prior to disposal in accordance with institutional safety protocols.

General Microwave-Assisted Synthesis of Isocyanides

3.1- General Microwave-Assisted Synthesis of Isocyanides on a 1.0 mmol scale.

A mixture of the primary amine (1.2 mmol) and chloroform (1.0 mmol) were combined with sodium hydroxide (4.0 mmol, 0.3 mL of distilled water) and Span 80® (0.02 mmol) as a biodegradable phase-transfer catalyst, in a 5 mL sealed glass tube. The biphasic mixture was stirred magnetically and heated under microwave irradiation at 80 °C for 30 min. After cooling to room temperature, the reaction mixture was adsorbed onto silica gel (10 g, 230–400 mesh) and directly loaded onto a prepacked silica column (230–400 mesh). Purification using a heptane/2-methyltetrahydrofuran (8:2) eluent afforded the corresponding isocyanide in 43–86% yield. Products were characterized by NMR data, which were consistent with the expected structures.

3.2- General Microwave-Assisted Synthesis of Isocyanides on a 10 mmol scale.

In a 20 mL sealed glass tube, the corresponding primary amine (12 mmol) and chloroform (10 mmol) were combined with sodium hydroxide (40 mmol, 3 mL of distilled water) and Span 80® (0.2 mmol) as a biodegradable phase-transfer catalyst. The biphasic mixture was stirred magnetically and heated under microwave irradiation at 80 °C for 30 min. After cooling to room

temperature, the reaction mixture was adsorbed onto silica gel (20 g, 230–400 mesh) and directly loaded onto a prepacked silica column (230–400 mesh). Purification using a heptane/2-methyltetrahydrofuran (8:2) eluent afforded the corresponding isocyanide in 59–96% yield. Products were characterized by NMR data, consistent with the expected structures.

Analytical Data

The references listed below correspond to previously described isocyanides and are provided for comparative purposes regarding analytical data (NMR), reported yields, and synthetic methodologies.

(I-1) Methyl isocyanoacetate.[55] ^{1}H NMR (600 MHz, $CDCl_3$) δ: 4.26 (s, 2H), 3.84 (s, 3H); ^{13}C NMR (151 MHz, $CDCl_3$) δ: 164.4, 161.4, 53.4, 43.4 (t, *J* = 6.2 Hz). Yield 88%; 872.0 mg. HRESI-MS (ESI-QTOF) *m/z* calculated for $C_4H_6NO_2^+$ $[M+H]^+$: 100.0393, found $[M+H]^+$: 100.0393. Purified product: colorless liquid.

(I-2) Methyl 3-isocyanopropanoate.[56] ^{1}H NMR (600 MHz, $CDCl_3$) δ: 3.72–3.71 (m, 3H), 3.68–3.65 (m, 2H), 2.73–2.69 (m, 2H); ^{13}C NMR (151 MHz, $CDCl_3$) δ: 169.7, 157.2 (t, *J* = 4.2 Hz), 52.1, 37.0 (t, *J* = 7.3 Hz), 33.8. Yield 80%; 905.0 mg. HRMS (ESI-QTOF) *m/z* calculated for $C_5H_8NO_2^+$ $[M+H]^+$: 114.0550, found $[M+H]^+$: 114.0551. Purified product: colorless liquid.

(I-3) Isopropyl isocyanide.[55] ^{1}H NMR (600 MHz, $CDCl_3$): δ 3.79–3.74 (m, 1H), 1.38–1.37 (s, 6H); ^{13}C NMR (151 MHz, $CDCl_3$): δ 153.6, 45.7, 23.4. Yield 70%; 483.7 mg. HRESI-MS (ESI-QTOF) *m/z* calculated for $C_4H_{10}NO^+$ $[M+H+H_2O]^+$: 88.0757, found $[M+H+H_2O]^+$: 88.0755. Purified product: colorless liquid.

(I-4) Isobutyl isocyanide.[55] ^{1}H NMR (600 MHz, $CDCl_3$) δ: 3.38–3.35 (m, 2H), 1.78–1.72 (m, 1H), 0.91 (d, *J* = 6.6 Hz, 6H); ^{13}C NMR (151 MHz, $CDCl_3$) δ: 155.8 (t, *J* = 4.4 Hz), 46.1, 24.9, 21.7. Yield 72%; 598.6 mg. HRESI-MS (ESI-QTOF) *m/z* calculated for $C_5H_{12}NO^+$ $[M+H+H_2O]^+$: 102.0913, found $[M+H+H_2O]^+$: 102.0912. Purified product: colorless liquid.

(I-5) 1-Isocyano-3-methylbutane.[57] ^{1}H NMR (600 MHz, $CDCl_3$) δ: 3.38–3.35 (m, 2H), 1.78-1.72 (m, 1 H), 1.55–1.52 (m, 2H), 0.91 (m, 6H); ^{13}C NMR (151 MHz, $CDCl_3$) δ: 157.5 (t, *J* = 5.8 Hz), 34.9 (t, *J* = 6.4 Hz), 27.6, 21.7. Yield 91%; 884.2 mg. HRESI-MS (ESI-QTOF) *m/z* calculated for $C_6H_{14}NO^+$ $[M+H+H_2O]^+$: 116.1075, found $[M+H+H_2O]^+$: 116.1073. Purified product: colorless liquid.

(I-6) tert-Butyl isocyanide.[55] ^{1}H NMR (600 MHz, $CDCl_3$) δ: 1.46 [s, 9H]; ^{13}C NMR (151 MHz, $CDCl_3$): δ 152.4; 30.5. Yield 59%; 490.5 mg. HRESI-MS (ESI-QTOF) *m/z* calculated for $C_5H_{12}NO^+$ $[M+H+H_2O]^+$: 102.0913, found $[M+H+H_2O]^+$: 102.0911. Purified product: colorless liquid.

(I-7) Butyl isocyanide.[55] ^{1}H NMR (600 MHz, $CDCl_3$) δ: 3.40–3.37 [m, 2H], 1.68–1.64 [m, 2H], 1.50–1.45 [m, 2H], 0.95 [t, 3H, *J*= 7,3 Hz]; ^{13}C NMR (151 MHz, $CDCl_3$) δ: 155.5, 41.2 (t, *J* = 6.5 Hz), 30.8, 19.5, 13.1. Yield 80%; 665.1 mg. HRESI-MS (ESI-QTOF) *m/z* calculated for $C_5H_{12}NO^+$ $[M+H+H_2O]^+$: 102.0913, found $[M+H+H_2O]^+$: 102.0912. Purified product: colorless liquid.

(I-8) Amyl isocyanide.[55] ^{1}H NMR (600 MHz, $CDCl_3$) δ 3.31–3.28 (m, 2H), 1.60–1.54 (m, 2H), 1.41–1.35 (m, 2H), 0.86 (t, *J* = 7.4 Hz, 3H); ^{13}C NMR (151 MHz, $CDCl_3$) δ 155.7, 41.2 (t, *J* = 6.4 Hz), 30.9, 21.4, 19.4, 12.5. Yield 84%, 816.2 mg. HRESI-MS (ESI-QTOF) *m/z* calculated for $C_6H_{14}NO^+$ $[M+H+H_2O]^+$: 116.1070, found $[M+H+H_2O]^+$: 116.1071. Purified product: colorless liquid.

(I-9) 1-Isocyanodecane.[55] ^{1}H NMR (600 MHz, $CDCl_3$) δ: 3.34 (t, *J* = 6.8 Hz, 2H), 1.66–1.63 (m, 2H), 1.41 – 1.38 (m, 2H), 1.27–1.24 (m, 12H), 0.86–0.84 (m, 3H); ^{13}C NMR (151 MHz, $CDCl_3$) δ: 155.5,

41.4 (t, J = 6.3 Hz), 31.7, 29.4, 29.3, 29.2, 29.1, 28.9, 28.5, 26.1, 13.9. Yield 89%; 1489.0 mg. HRESI-MS (ESI-QTOF) *m/z* calculated for $C_{11}H_{24}NO^+$ $[M+H+H_2O]^+$: 186.1852, found $[M+H+H_2O]^+$: 186.1851. Purified product: pale yellow pasty material.

(I-10) Dodecyl isocyanide.[55] ^1H NMR (600 MHz, $CDCl_3$) δ: 3.40 – 3.38 [m, 2H], 1.72–1.66 [m, 2H], 1.47–1.42 [m, 2H], 1.34–1.26 [m, 16H], 0.91–0.89 [m, 3H]; NMR ^{13}C (151 MHz, $CDCl_3$) δ: 155.6, 41.6, 31.9, 29.6, 29.5, 29.4, 29.3, 29.1, 28.6, 26.3, 22.8, 14.1. Yield 92%; 1797.2 mg. HRESI-MS (ESI-QTOF) *m/z* calculated for $C_{13}H_{28}NO^+$ $[M+H+H_2O]^+$: 214.2165, found $[M+H+H_2O]^+$: 214.2163. Purified product: pale yellow pasty material.

(I-11) Octadecyl isocyanide.[55] ^1H NMR (600 MHz, $CDCl_3$) δ: 3.41–3.38 [m, 2H], 1.71–1.68 [m, 2H], 1.48–1.43 [m, 2H], 1.35–1.28 [m, 28H], 0.91–0.89 [m, 3H]; NMR ^{13}C (151 MHz, $CDCl_3$) δ: 155.6, 41.6, 31.9, 29.7, 29.6, 29.5, 29.4, 29.1, 26.3, 22.8, 14.1. Yield 91%; 2543.6 mg. HRESI-MS (ESI-QTOF) *m/z* calculated for $C_{19}H_{40}NO^+$ $[M+H+H_2O]^+$: 298.3104, found $[M+H+H_2O]^+$: 298.3104. Purified product: light-brown paste.

(I-12) Cyclohexyl isocyanide.[55] ^1H NMR (600 MHz, $CDCl_3$) δ: 3.63–3.59 [m, 1H], 1.91–1.88 [m, 2H], 1.79–1.66 [m, 4H], 1.51–1.35 [m, 4H]; ^{13}C NMR (151 MHz, $CDCl_3$) δ: 153.9, 51.7, 32.7, 24.9, 22.7. Yield 87%; 949.8 mg. HRESI-MS (ESI-QTOF) *m/z* calculated for $C_7H_{14}NO^+$ $[M+H+H_2O]^+$: 128.1070, found $[M+H+H_2O]^+$: 128.1069. Purified product: colorless liquid.

(I-13) Phenyl isocyanide.[55] NMR ^1H (600 MHz, $CDCl_3$) δ: 7.44–7.40 (m, 5H); ^{13}C NMR (151 MHz, $CDCl_3$) δ: 163.8, 146.3, 129.3, 129.1, 126.3. Yield 88%; 907.5 mg. HRESI-MS (ESI-QTOF) *m/z* calculated for $C_7H_6N^+$ $[M+H]^+$: 104.0495, found $[M+H]^+$: 104.0495. Purified product: pale yellow liquid.

(I-14) (Isocyanomethyl)benzene.[58] ^1H NMR (600 MHz, $CDCl_3$) δ: 7.42–7.40 (m, 2H), 7.38–7.35 (m, 3H), 4.64 (s, 2H); ^{13}C NMR (151 MHz, $CDCl_3$) δ: 157.4 (t, J = 5.3 Hz), 132.1, 128.7, 128.1, 126.6, 45.2 (t, J = 7.3 Hz). Yield 95%; 1112.1 mg. HRESI-MS (ESI-QTOF) *m/z* calculated for $C_7H_7^+$ $[M+H-HCN]^+$: 91.0542, found $[M+H-HCN]^+$: 91.0542. Purified product: colorless liquid.

(I-15) 2-(Isocyanoethyl)benzene.[55] ^1H NMR (600 MHz, $CDCl_3$)) δ 7.43 – 7.22 (m, 5H), 3.61 (tt, J = 7.1, 1.9 Hz, 2H), 3.00 (tt, J = 7.1, 2.2 Hz, 2H); ^{13}C NMR (151 MHz, $CDCl_3$) δ 156.5 (t, J = 5.6 Hz), 136.6 (br t), 128.7, 128.7, 127.2, 42.9 (t, J = 6.6 Hz), 35.5 Yield 96%; 1259.3 mg. HRMS (ESI) *m/z* calculated for $C_8H_9^+$ [M + H – HCN]+: 105.0699; found, 105.0698. Purified product: yellow pale liquid.

(I-16) 2-Isocyanoaniline.[55] ^1H NMR (600 MHz, $CDCl_3$) δ: 7.24–7.18 (m, 2H), 6.80–6.71 (m, 2H), 4.19 (bs, 2H); ^{13}C NMR (151 MHz, $CDCl_3$) δ: 167.1, 142.7, 130.3, 126.7, 118.1, 115.5. Yield 73%; 86.24 mg. HRESI-MS (ESI-QTOF) *m/z* calculated for $C_7H_7N_2^+$ $[M+H]^+$: 119.0604, found $[M+H]^+$: 119.0602. Purified product: pale yellow liquid.

(I-17) 1-Isocyano-2-methylbenzene.[59] ^1H NMR (600 MHz, $CDCl_3$) δ: 7.35–7.33 (m, 1 H), 7.30–7.28 (m, 2H), 7.22–7.20 (m, 1H), 2.43 (s, 3H); ^{13}C NMR (151 MHz, $CDCl_3$) δ: 165.5 (t, J = 5.8 Hz), 134.9, 130.5, 129.2, 126.6, 126.5, 18.5. Yield 78%; 91.38 mg. HRESI-MS (ESI-QTOF) *m/z* calculated for $C_8H_8N^+$ $[M+H]^+$: 118.0651, found $[M+H]^+$: 118.0652. Purified product: colorless liquid.

(I-18) 1-Isocyano-3-methylbenzene.[59] ^1H NMR (600 MHz, $CDCl_3$) δ: 7.25–7.23 (m, 1H), 7.18–7.14 (m, 3H), 2.36 (s, 3H); ^{13}C NMR (151 MHz, $CDCl_3$) δ: 163.3 (t, J = 5.6 Hz), 139.6, 130.1, 129.1, 126.8, 123.4, 21.1. Yield 80%; 93.72 mg. HRESI-MS (ESI-QTOF) *m/z* calculated for $C_8H_8N^+$ $[M+H]^+$: 118.0651, found $[M+H]^+$: 118.0652. Purified product: colorless liquid.

(I-19) 1-Isocyano-4-methylbenzene.[59] ^{1}H NMR (600 MHz, $CDCl_3$) δ: 7.26 (d, *J* = 8.2 Hz, 2H), 7.18 (d, *J* = 8.2 Hz, 2H), 2.37 (s, 3H); ^{13}C NMR (151 MHz, $CDCl_3$) δ: 163.1 (t, *J* = 5.6 Hz), 139.7, 129.9, 126.1, 124.0 (t, *J* = 13.8 Hz), 21.3. Yield 85%; 99.58 mg. HRESI-MS (ESI-QTOF) *m/z* calculated for $C_8H_8N^+$ $[M+H]^+$: 118.0651, found [M+H]+: 118.0652. Purified product: colorless liquid.

(I-20) 2-Isocyanopyridine.[58] ^{1}H NMR (600 MHz, $CDCl_3$) δ: 8.50 (dd, *J* = 4.9, 1.1 Hz, 1H), 7.82 (td, *J* = 7.8, 1.9 Hz, 1H), 7.40–7.35 (m, 2H); ^{13}C NMR (151 MHz, $CDCl_3$) δ: 163.8, 149.8, 140.4 (t, *J* = 17.2 Hz), 138.7, 124.7, 121.4. Yield 45%; 53.16 mg. HRESI-MS (ESI-QTOF) *m/z* calculated for $C_6H_7N_2O^+$ $[M+H+H_2O]^+$: 123.0558, found $[M+H+H_2O]^+$: 123.0560. Purified product: pale yellow liquid.

(I-21) 5-Isocyano-2-methylpyridine.[55] ^{1}H NMR (600 MHz, $CDCl_3$) δ: 8.56 (d, *J* = 2.4 Hz, 1H), 7.57 (dd, *J* = 8.3, 2.4 Hz, 1H), 7.21 (d, *J* = 8.3 Hz, 1H), 2.59 (s, 3H); ^{13}C NMR (151 MHz, $CDCl_3$) δ: 168.0, 159.5, 146.7, 133.5, 123.4, 24.4. Yield 43%, 44.77 mg. HRESI-MS (ESI-QTOF) *m/z* calculated for $C_7H_9N_2O^+$ $[M+H+H_2O]^+$: 137.0715, found $[M+H+H_2O]^+$: 137.0716. Purified product: pale yellow liquid.

Calculation of Green Chemistry Metrics

Assessment of the energy requirements of the processes

The energy consumed during the process was calculated using the equation: E = (P × t)/1000, where E is the energy in kWh, P is the power in watts (W), and t is the time in hour (h).
For the energy consumption calculations, the synthesis of the formamide intermediate was not considered, as this step typically requires approximately 12 h. Inclusion of this step would further increase the difference between the conventional heating and microwave-assisted methodologies.

For a system operating at a maximum power of 300 W (microwave reactor) over a duration of 30 minutes (i.e., 0.5 h), the total energy consumption is:

$$E = (300\ W \times 0.5\ h)/1000 = 0.15 kWh$$

For a laboratory heating mantle/reflux system operating at 600 W (magnetic stirrer) over a duration of 3 h, the total energy consumption is::

$$E = (20\ W \times 3\ h)/1000 = 1.8 kWh$$

Environmental factor (E-factor) Calculations:

The reaction conditions for the synthesis of phenethyl isocyanide (I-15) using $POCl_3$ were adapted from the literature [51–53]. During the calculation of the E-factors, neither the synthesis of the key intermediate (formamide) nor the amount of silica gel used in the purification step (flash column chromatography) was considered, since these data are generally not reported in the literature. However, if the typical amounts of silica gel employed in the reported methods, as well as the formamide preparation step, were considered, the E-factors of the first three literature reports would be even higher.

For the methodology described in this work, approximately 80 g of silica gel (100–200 mesh) was used for a 10 mmol scale reaction. As these parameters were excluded from the previously reported methodologies, they were likewise omitted from our calculations to ensure a consistent basis for comparison.

The calculations correspond to a simplified comparative E-factor estimation and do not represent a complete industrial life-cycle assessment.

The E-factor calculations were based on the following equation:

E-factor = total mass Kg (waste)/mass Kg (product)

Ingold, M.; López, G.V.; Porcal, W. Green conditions for Passerini three-component synthesis of tocopherol analogues. *ACS Sustain. Chem. Eng.* 2014, *2*, 1093–1097. https://doi.org/10.1021/sc5002116

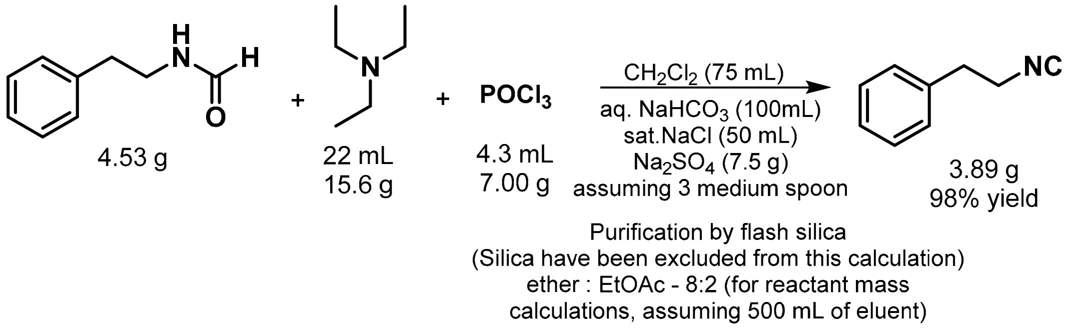

Total mass of process inputs:

Mass (N-(2-phenylethyl) formamide) = 4.53 g

Mass (Triethylamine) = 15.35 g

Mass ($POCl_3$) = 7.00 g

Mass (CH_2Cl_2 (assuming 90% recovery)): 75 ml x (1.33 g/ml) x 10% = 9.975 g

Mass (Aq. $NaHCO_3$, (calculated by weight) (100 ml)) = 106.7 g

Mass (Sat. NaCl sol. (calculated by weight): (50ml)) = 60.75 g

Purification by flash silica column solvent (8:2 ether/EtOAc) for 4.53 g assuming as 500 ml used and 90% recovery):

Mass (Ether (assuming as 90% recovery)): 400ml x (0.706 g/ml) x 10% = 28.24 g

Mass (EtOAc (assuming as 90% recovery)): 100 ml x (0.902 gm/ml) x 10% = 9.02 g

Mass (Na2SO4 used for drying (assuming two tea spoon)) = 5.0 g (Silica have been excluded from this calculation)

Total mass of Reactants waste: (4.53 g + 15.35 g + 7.00 g + 9.975 g + 106.7 g + 60.75 g + 28.24 g + 9.02 g + 5.0 g) = 246.565 g = 246.57 g

Amount of product = 3.89 g

E-Factor = Amount of waste/Amount of product = 246.57 g/3.89 g = 63.39

E-factor = 63.39

Grolla, A.A.; Podestà, V.; Chini, M.G.; Di Micco, S.; Vallario, A.; Genazzani, A.A.; Canonico, P.L.; Bifulco, G.; Tron, G.C.; Sorba, G.; Pirali, T. Synthesis, biological evaluation, and molecular docking of Ugi products containing a zinc-chelating moiety as novel inhibitors of histone deacetylases. *J. Med. Chem.* 2009, *52*, 2776–2785. https://doi.org/10.1021/jm801529c

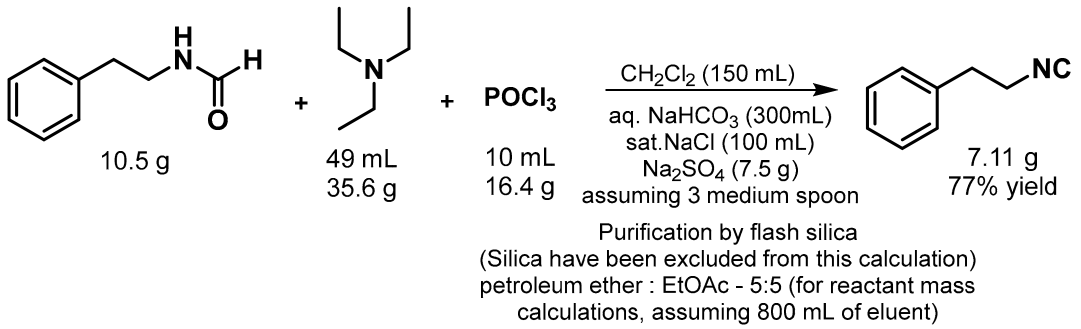

Total mass of process inputs:

Mass (N-(2-phenylethyl) formamide) = 10.5 g

Mass (Triethylamine) = 35.6 g

Mass (CH_2Cl_2 (assuming 90% recovery)): 150 ml x (1.33 g/ml) x 10% = 19.95 g

Mass (POCl3) = 16.40 g

Mass (Aq. NaHCO3, (calculated by weight) (3 x 100 ml)) = 320.1 g

Mass (Sat. NaCl sol. (calculated by weight): (100ml)) = 121.5 g

Purification by flash silica column solvent (5:5 ether/EtOAc) for 10.5 g assuming as 800 ml used and 90% recovery):

Mass (Pet. ether (assuming as 90% recovery)): 400ml x (0.64 g/ml) x 10% = 25.6 g

Mass (EtOAc (assuming as 90% recovery)): 400 ml x (0.902 gm/ml) x 10% = 36.08 g

Mass (Na2SO4 used for drying (assuming 3 tea spoon)) = 7.5 gm (Silica have been excluded from this calculation)

Total mass of Reactants waste: (10.5 gm + 35.6 gm + 19.95 gm + 16.40 gm + 320.1 gm + 121.5 g + 25.6 g + 36.08 g + 7.5 g) = 593.23 g

Amount of product = 7.11 g

E-Factor = Amount of waste/Amount of product = 593.23 g/7.11 g = 83.44

E-factor = 83.44

Goldeman, W.; Nasulewicz-Goldeman, A. Synthesis and biological evaluation of aminomethylidenebisphosphonic derivatives of β-arylethylamines. Tetrahedron 2015, 71, 3282–3289. https://doi.org/10.1016/j.tet.2015.03.112

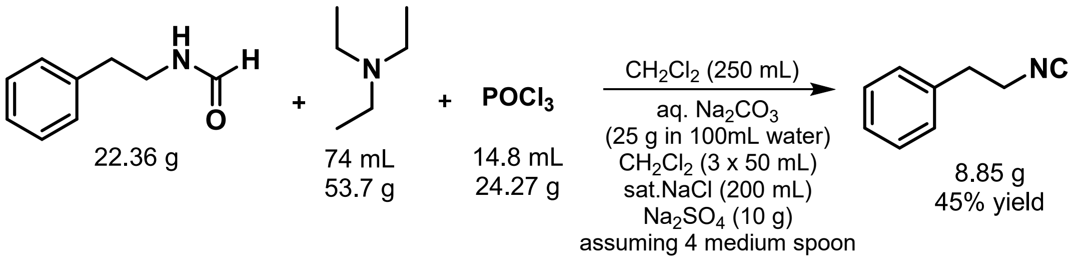

Purification was performed by bulb-to-bulb distillation.

Total mass of process inputs:

Mass (N-(2-phenylethyl) formamide) = 22.36 g

Mass (Triethylamine) = 53.7 g

Mass (CH_2Cl_2 (assuming 90% recovery)): 400 ml x (1.33 g/ml) x 10% = 54.37 g

Mass (POCl3) = 24.27 g

Mass (Na2CO3 Mass) = 25.0 g

Mass (Water (100 ml)) = 100.0 g

Mass (Sat. NaCl sol. (calculated by weight): (200ml)) = 243.0 g

Mass (Na2SO4 used for drying (assuming 4 tea spoon)) = 10.0 g

Total mass of Reactants waste: (22.36 g + 53.7 g + 54.37 g + 24.27 g + 25.0 g + 100 g + 243.0 g + 10.0 g) = 532.7 g

Amount of product = 8.85 g

E-Factor = Amount of waste/Amount of product = 532.7 g/8.85 g = 60.19

E-factor = 60.19

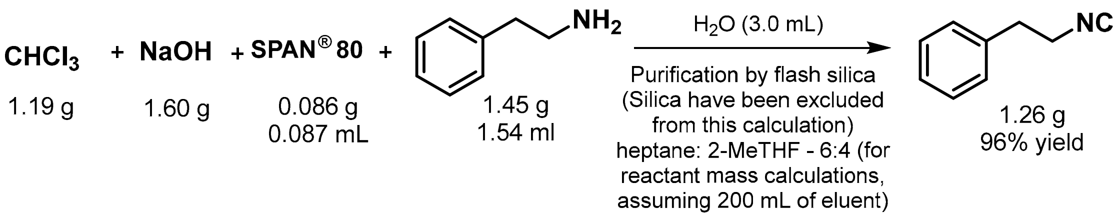

Total mass of process inputs:

Mass (Chloroform) = 1.19 g

Mass (Sodium hydroxide) = 1.6 g

Mass (SPAN®80) = 0.086 g

Mass (Amine) = 1.45 g

Mass (H_2O) = 3.0 g

Amount of total amount of Purification:

Mass (Heptane (assuming 90% recovery)): 160 ml x (0.684 g/ml) x 10% = 10.94 g.

Mass (2-MeTHF (assuming 90% recovery)): 40 ml x (0.853 g/ml) x 10% = 3.41 g.

Silica (80.0 g) have been excluded from this calculation, as in all above mention.

Mass (waste): 1.19 + 1.6 + 0.086 + 1.45 + 3.0 + 10.94 + 3.41 = 21.68 g

Mass (Product) = 1.26 g

E-Factor = Amount of waste/Amount of product = 21.68 g/ 1.26 g = 17.20

E-factor = 17.20

Spectra NMR (^{1}H NMR and ^{13}C NMR)

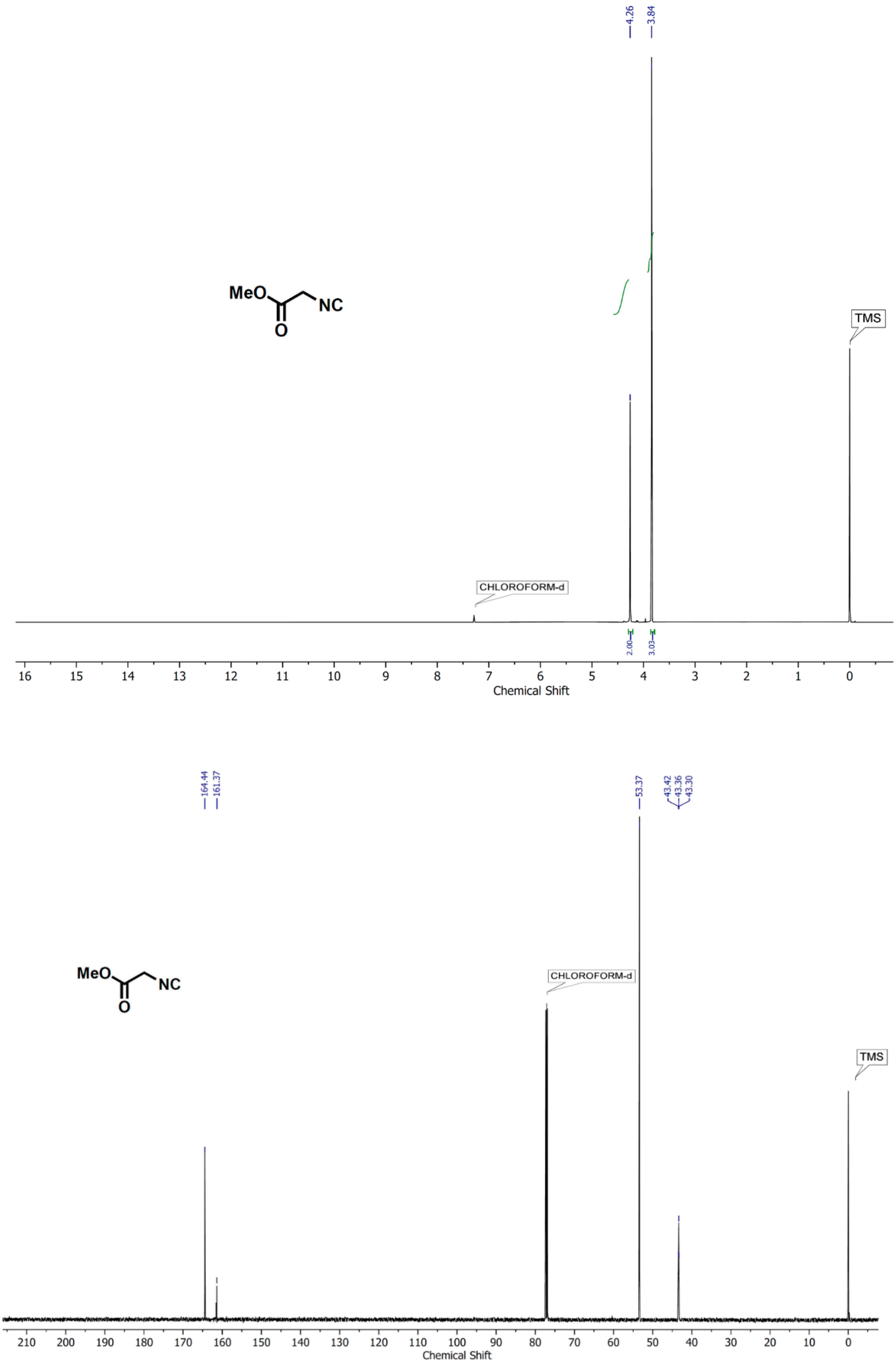

Figure S1. ^{1}H NMR (600 MHz, $CDCl_3$) and ^{13}C NMR (151 MHz, $CDCl_3$) of compound I-1

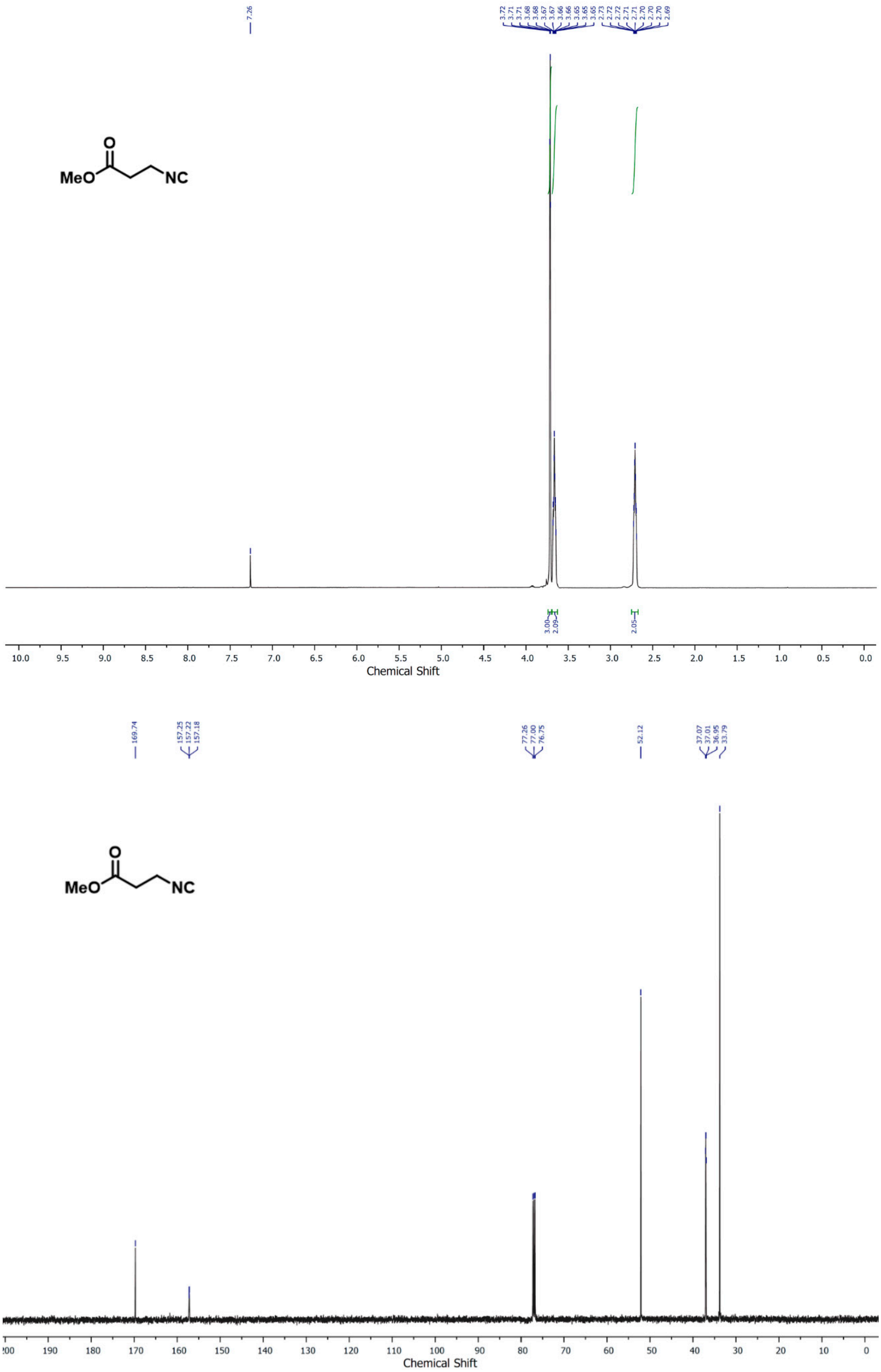

Figure S2. ^{1}H NMR (600 MHz, $CDCl_3$) and ^{13}C NMR (151 MHz, $CDCl_3$) of compound I-2

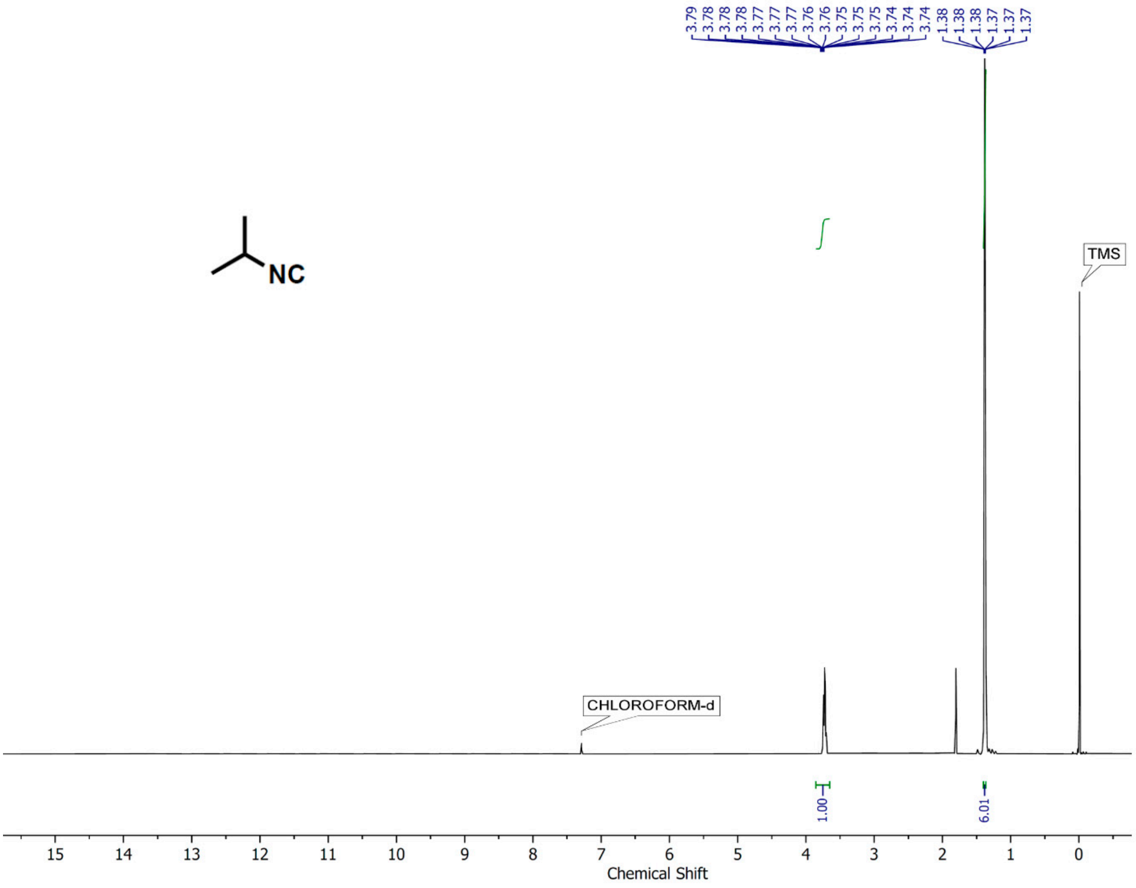

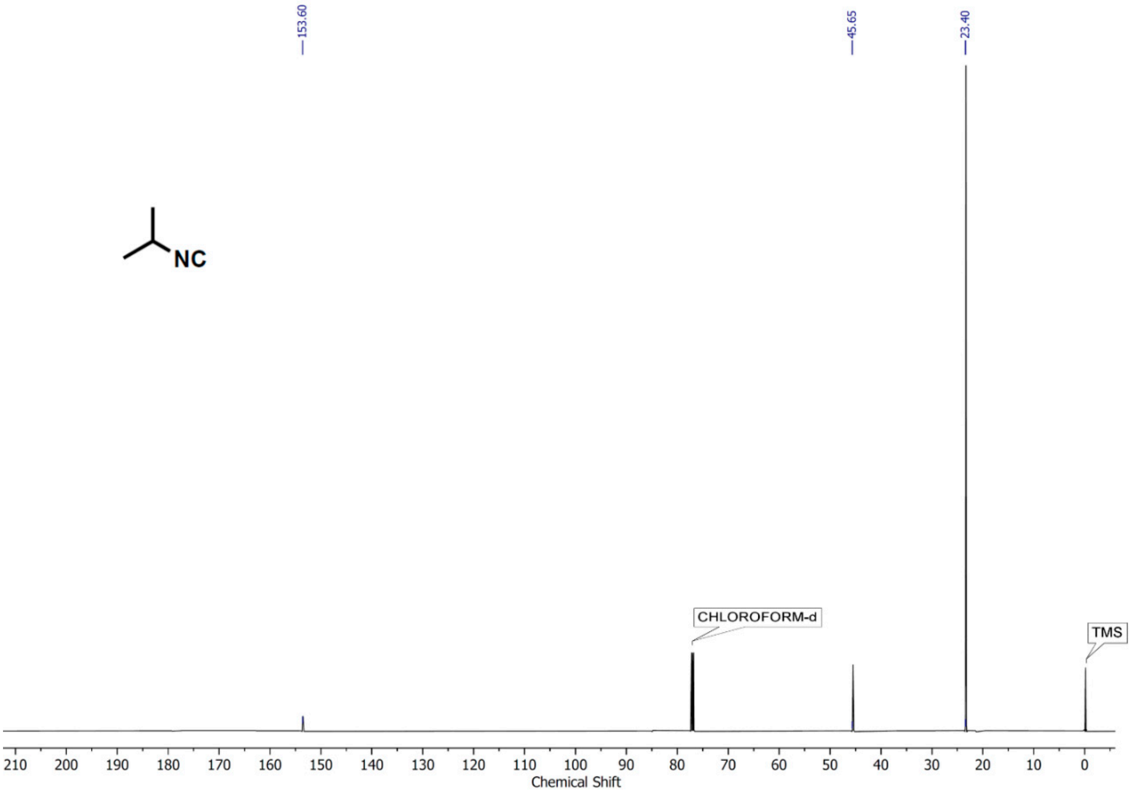

Figure S3. ^{1}H NMR (600 MHz, $CDCl_3$) and ^{13}C NMR (151 MHz, $CDCl_3$) of compound I-3

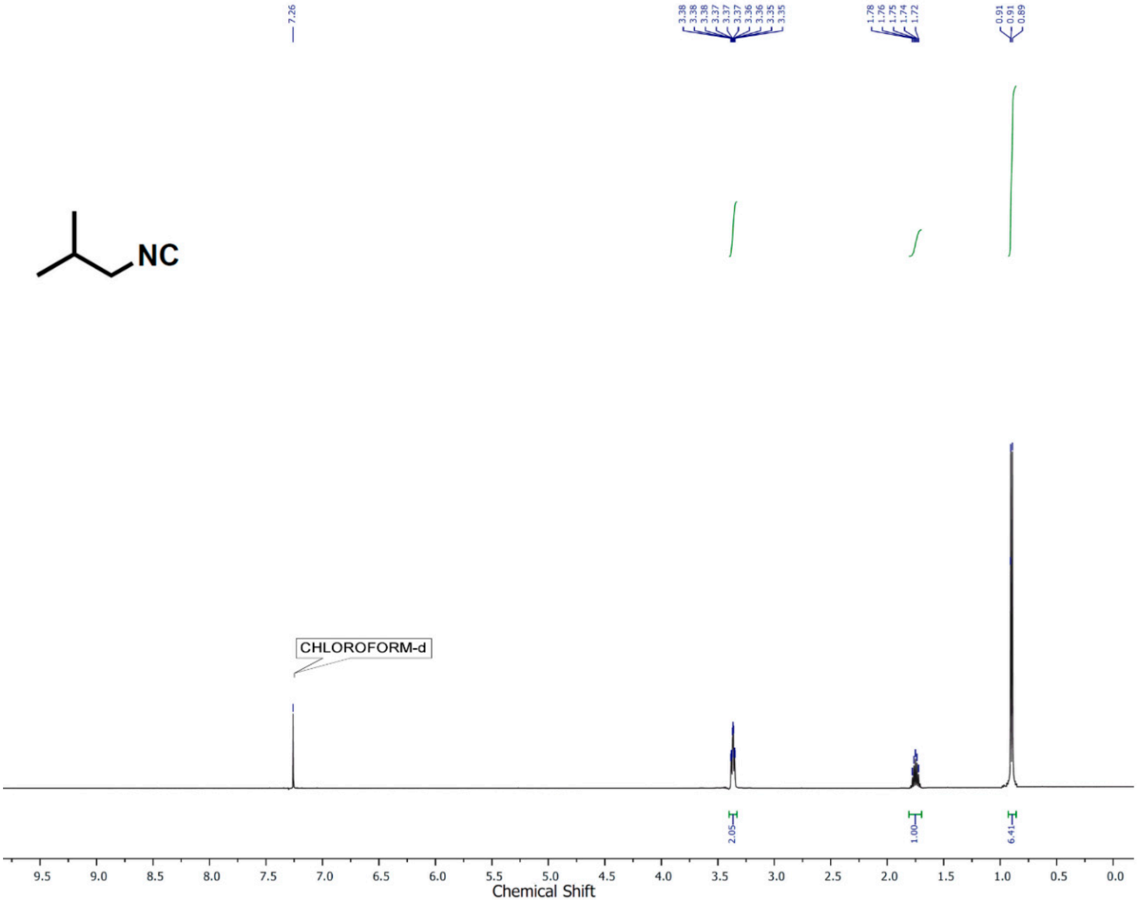

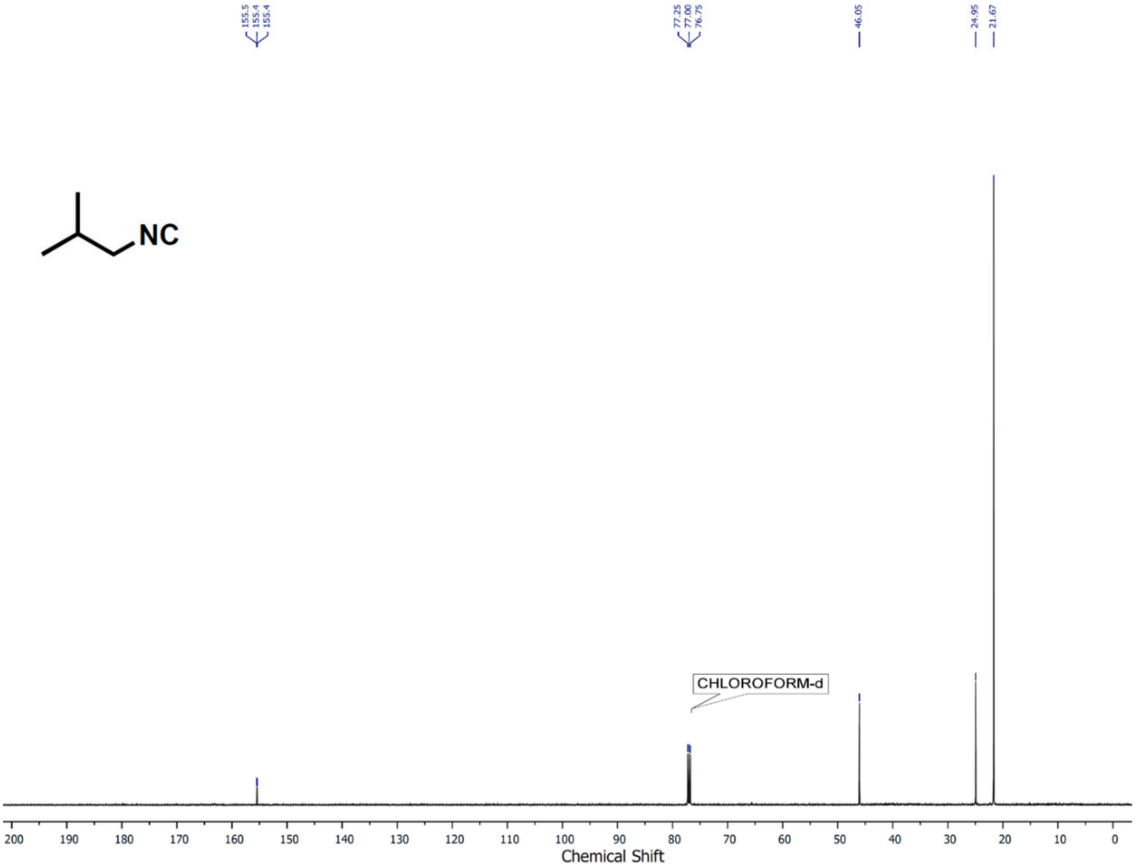

Figure S4. ^{1}H NMR (600 MHz, $CDCl_3$) and ^{13}C NMR (151 MHz, $CDCl_3$) of compound I-4

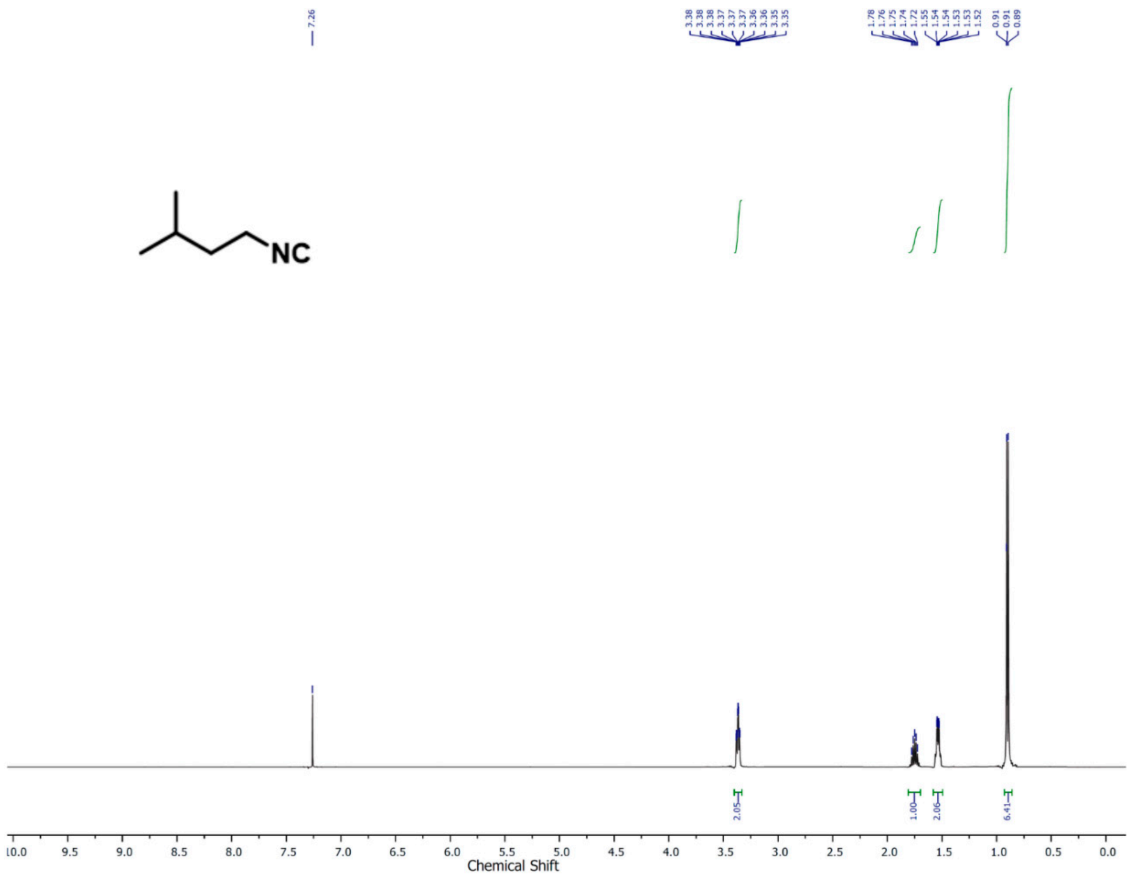

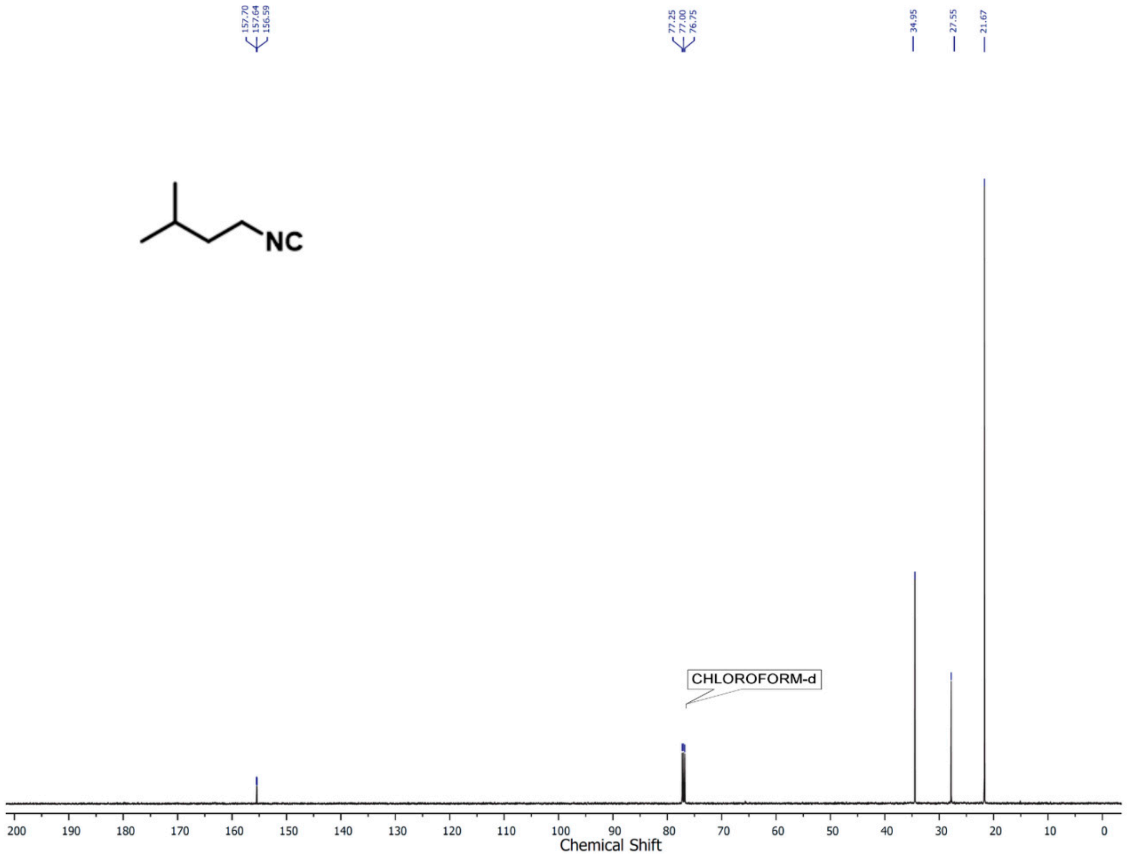

Figure S5. ^{1}H NMR (600 MHz, $CDCl_3$) and ^{13}C NMR (151 MHz, $CDCl_3$) of compound I-5

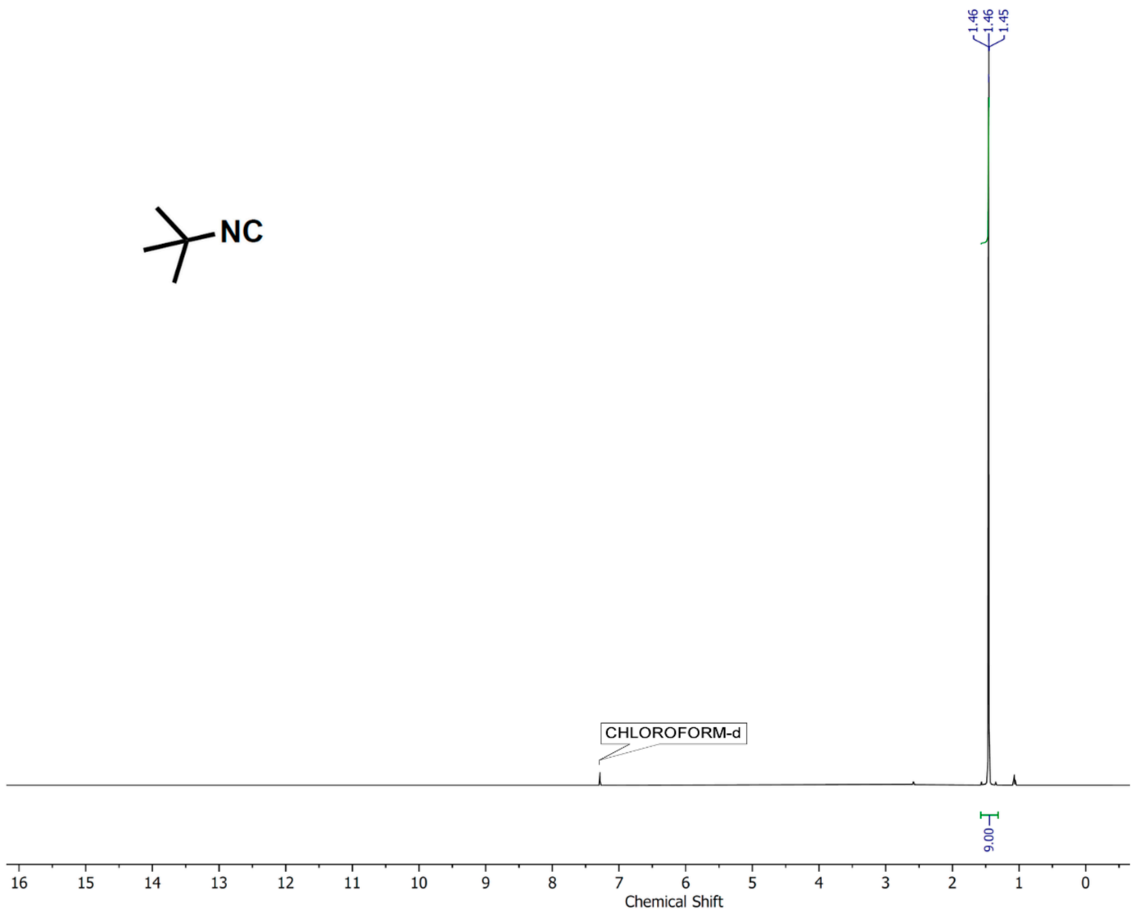

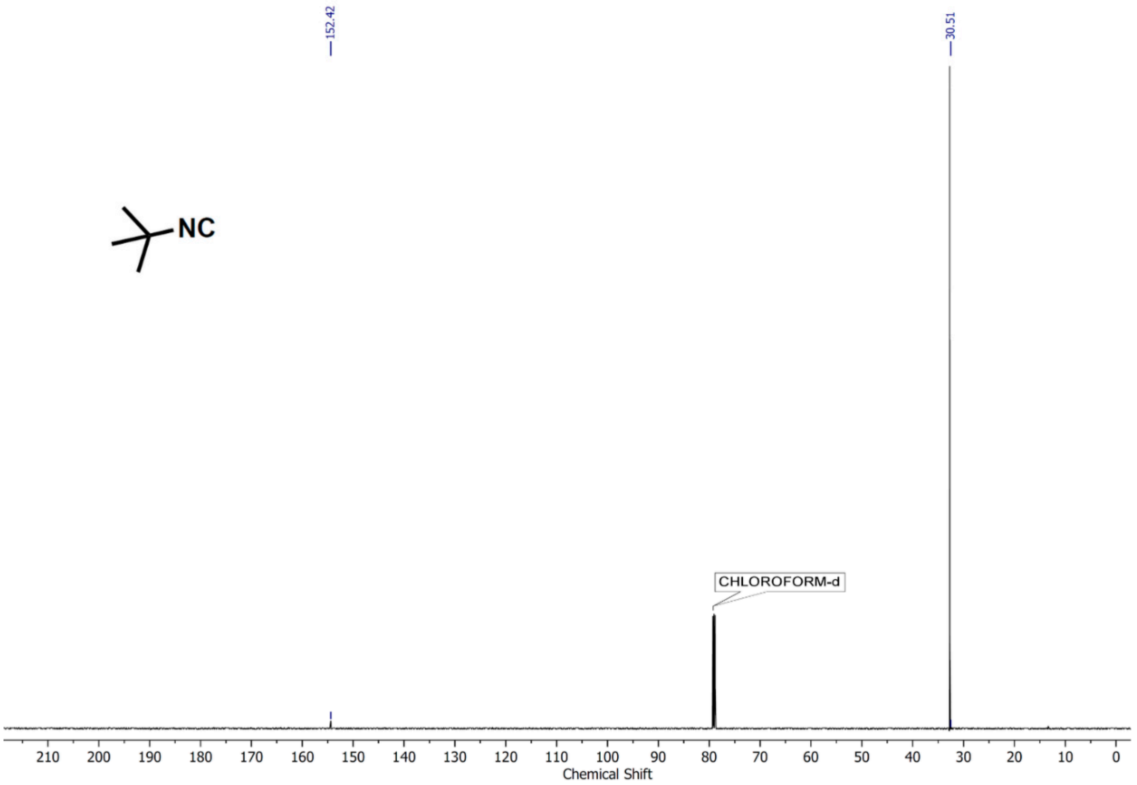

Figure S6. ^{1}H NMR (600 MHz, $CDCl_3$) and ^{13}C NMR (151 MHz, $CDCl_3$) of compound I-6

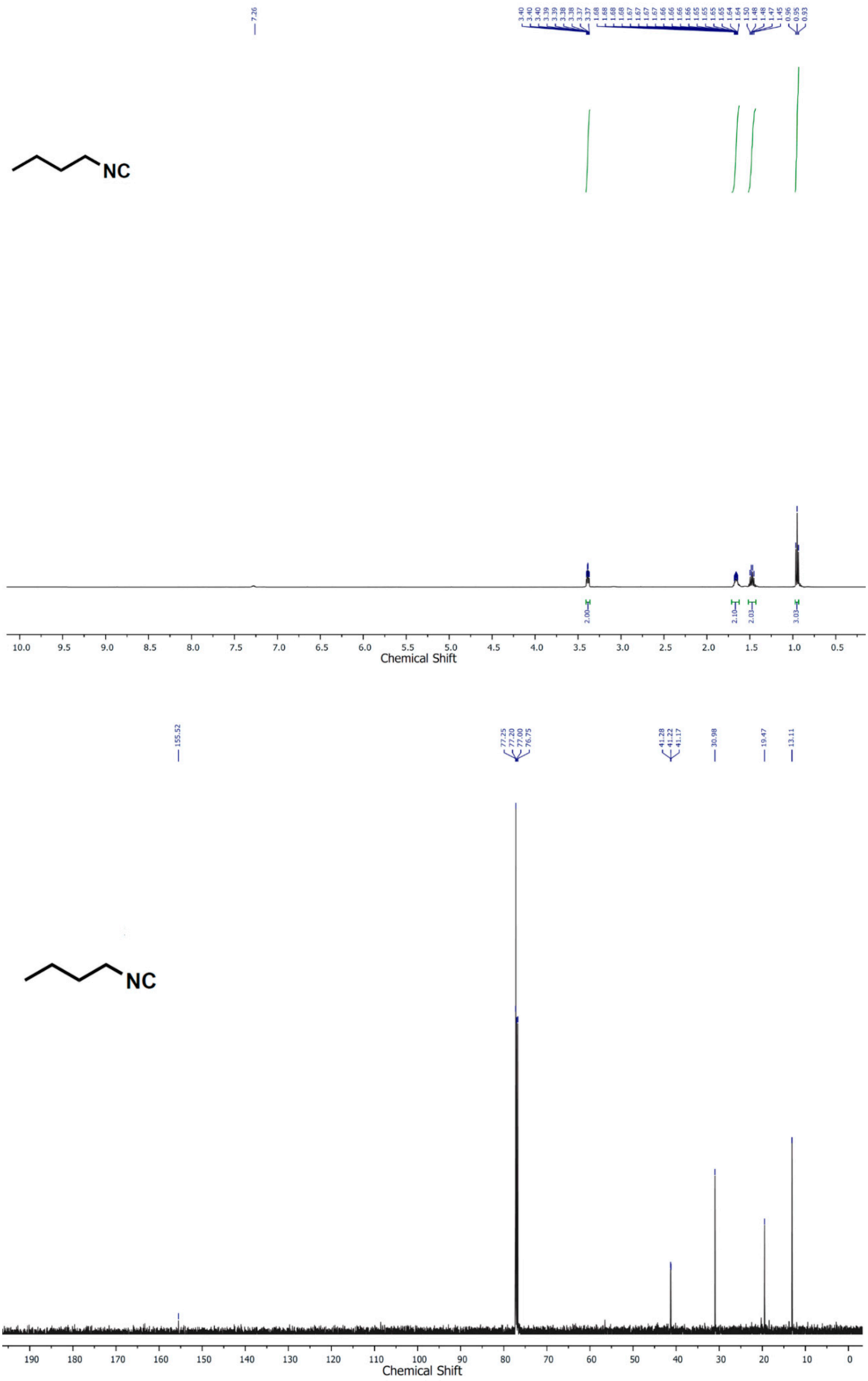

Figure S7. ^{1}H NMR (600 MHz, $CDCl_3$) and ^{13}C NMR (151 MHz, $CDCl_3$) of compound I-7

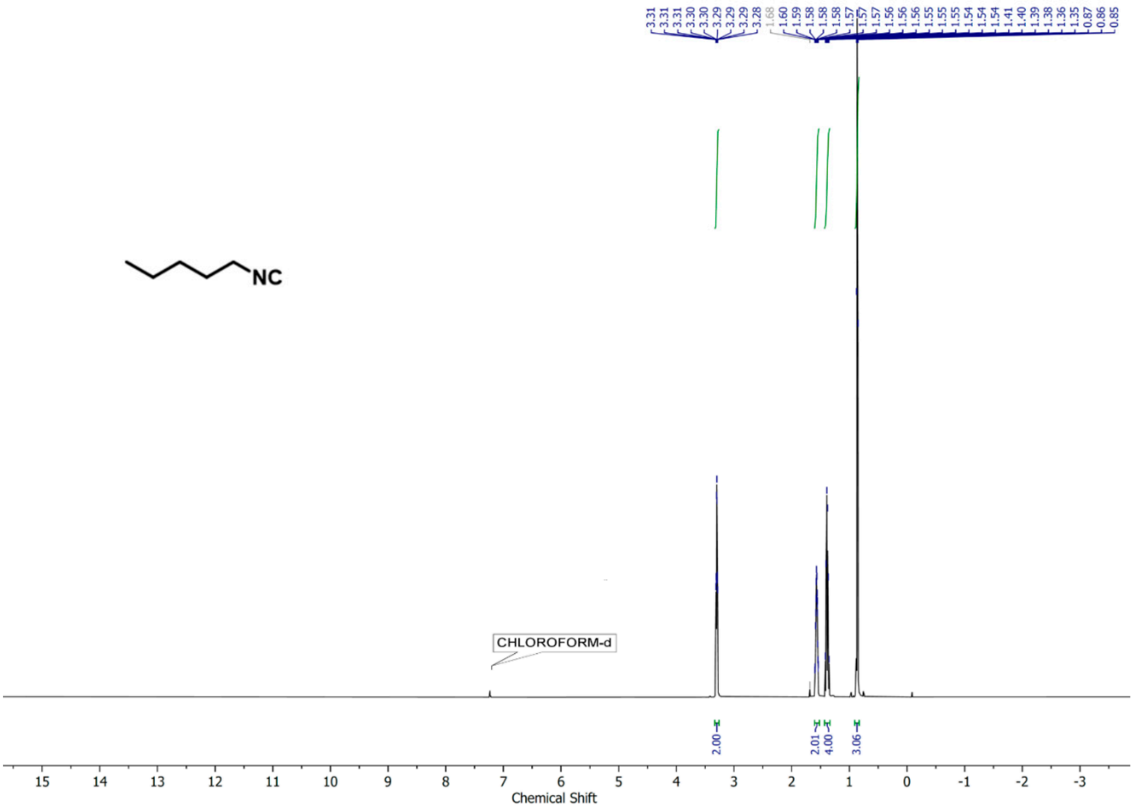

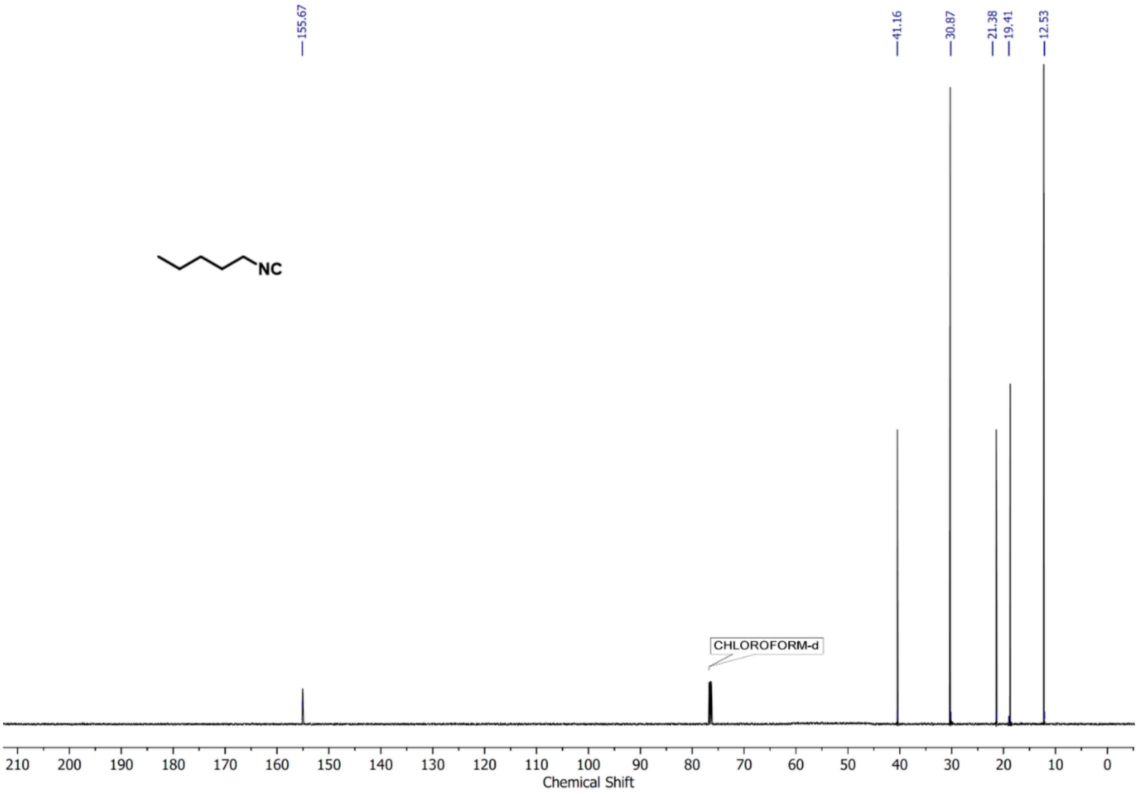

Figure S8. ^{1}H NMR (600 MHz, $CDCl_3$) and ^{13}C NMR (151 MHz, $CDCl_3$) of compound I-8

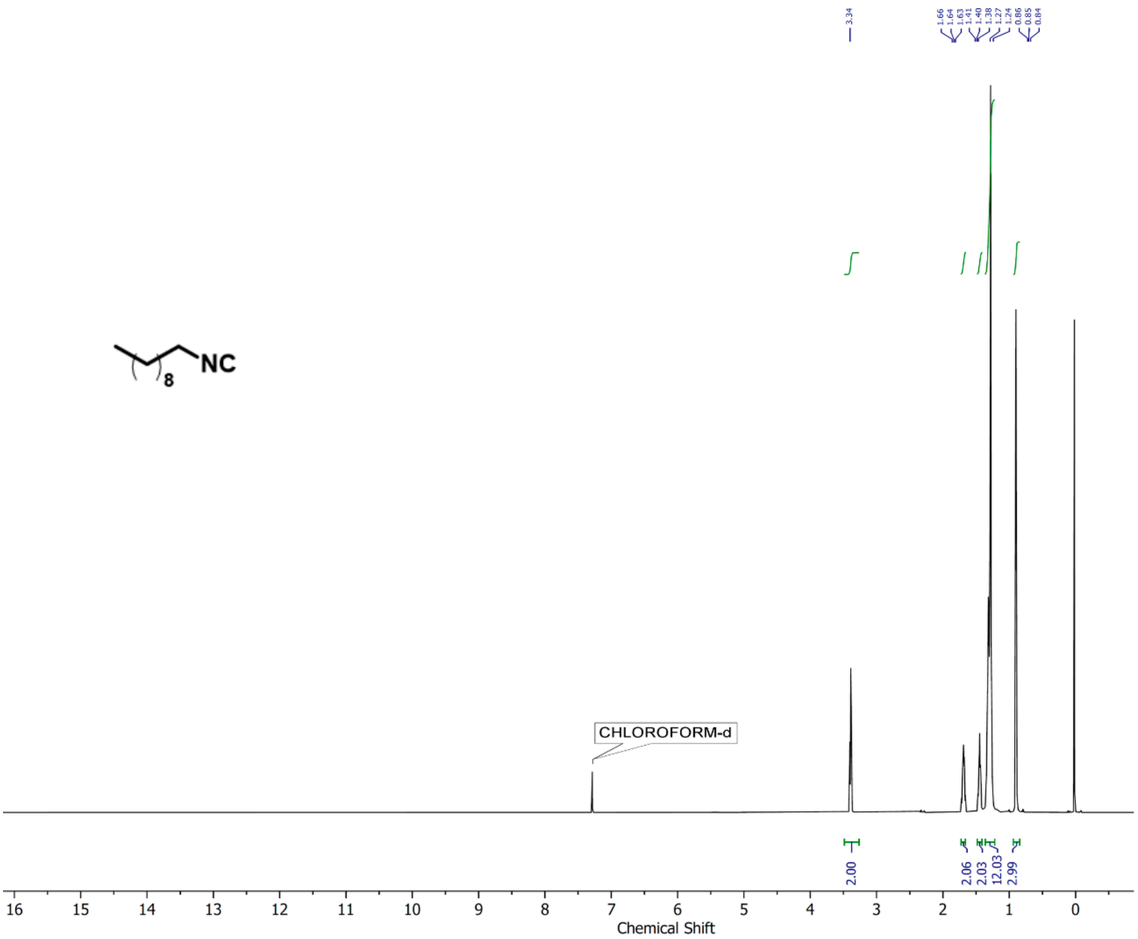

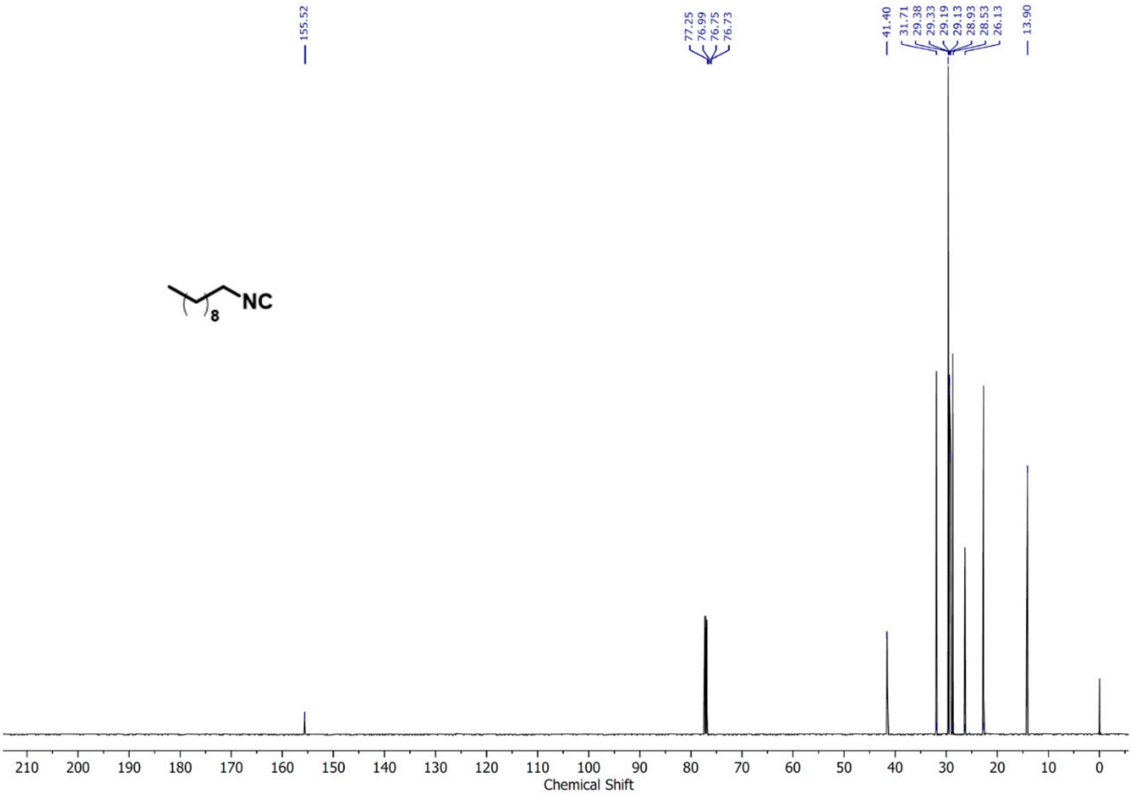

Figure S9. ^{1}H NMR (600 MHz, $CDCl_3$) and ^{13}C NMR (151 MHz, $CDCl_3$) of compound I-9

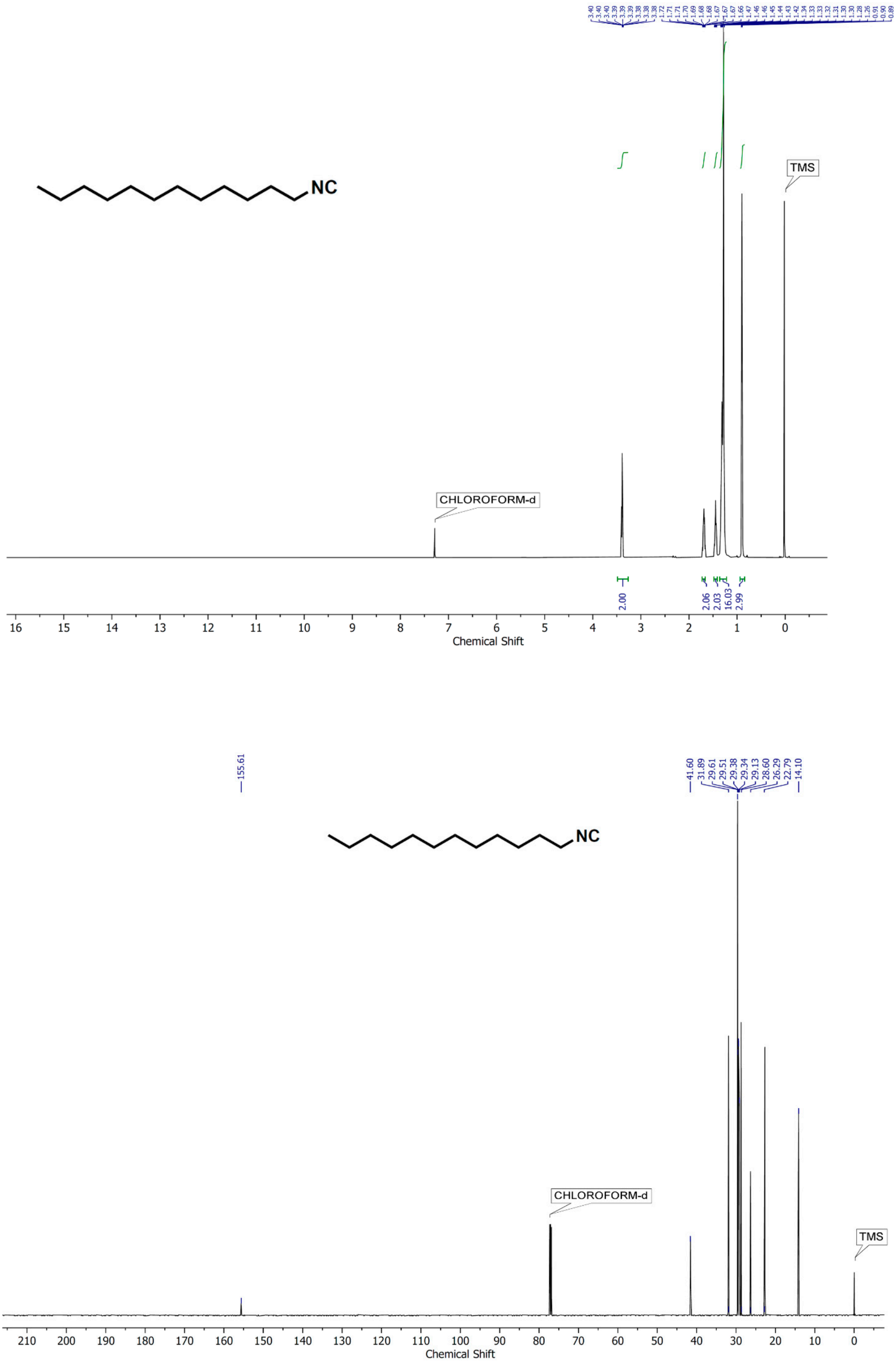

Figure S10. ^{1}H NMR (600 MHz, $CDCl_3$) and ^{13}C NMR (151 MHz, $CDCl_3$) of compound I-10

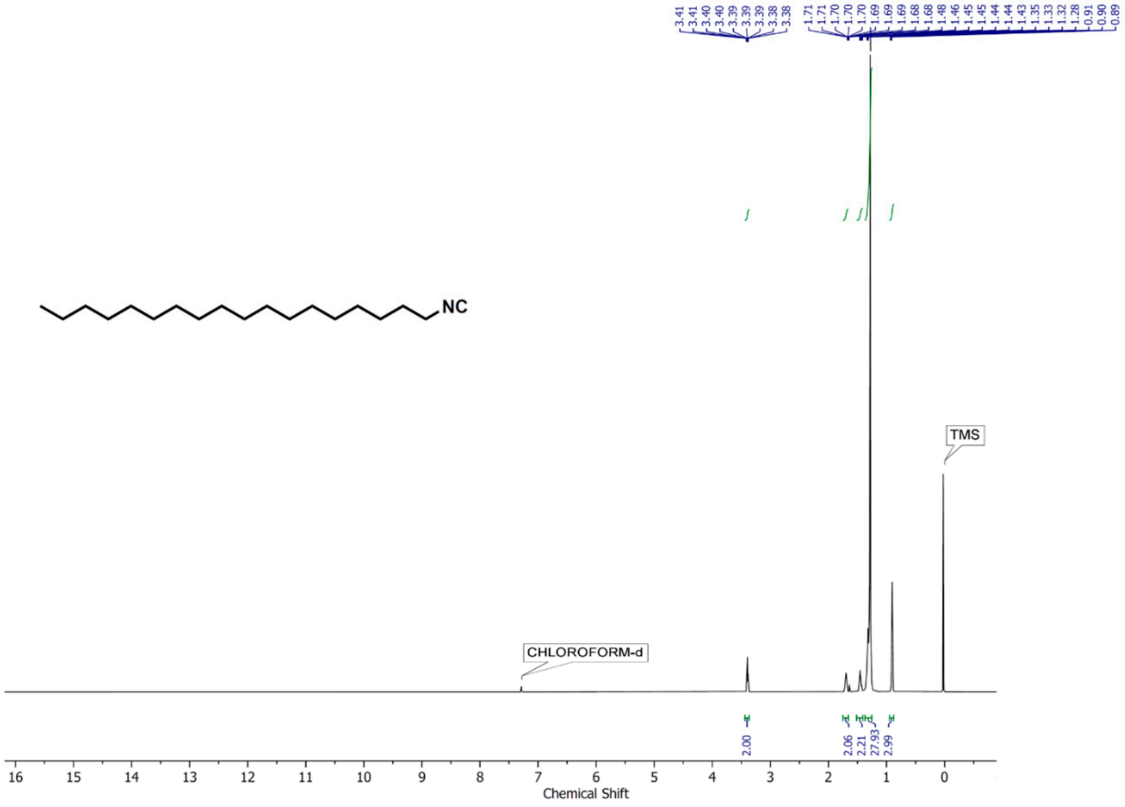

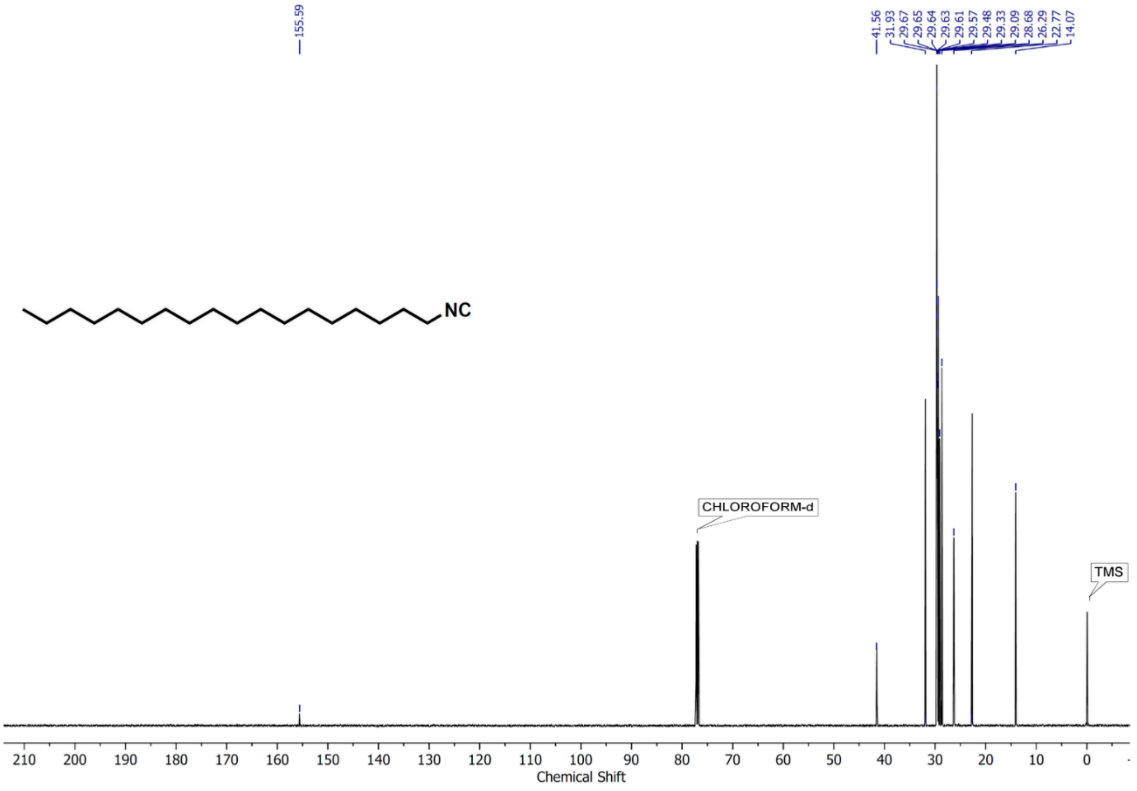

Figure S11. ^{1}H NMR (600 MHz, $CDCl_3$) and ^{13}C NMR (151 MHz, $CDCl_3$) of compound I-11

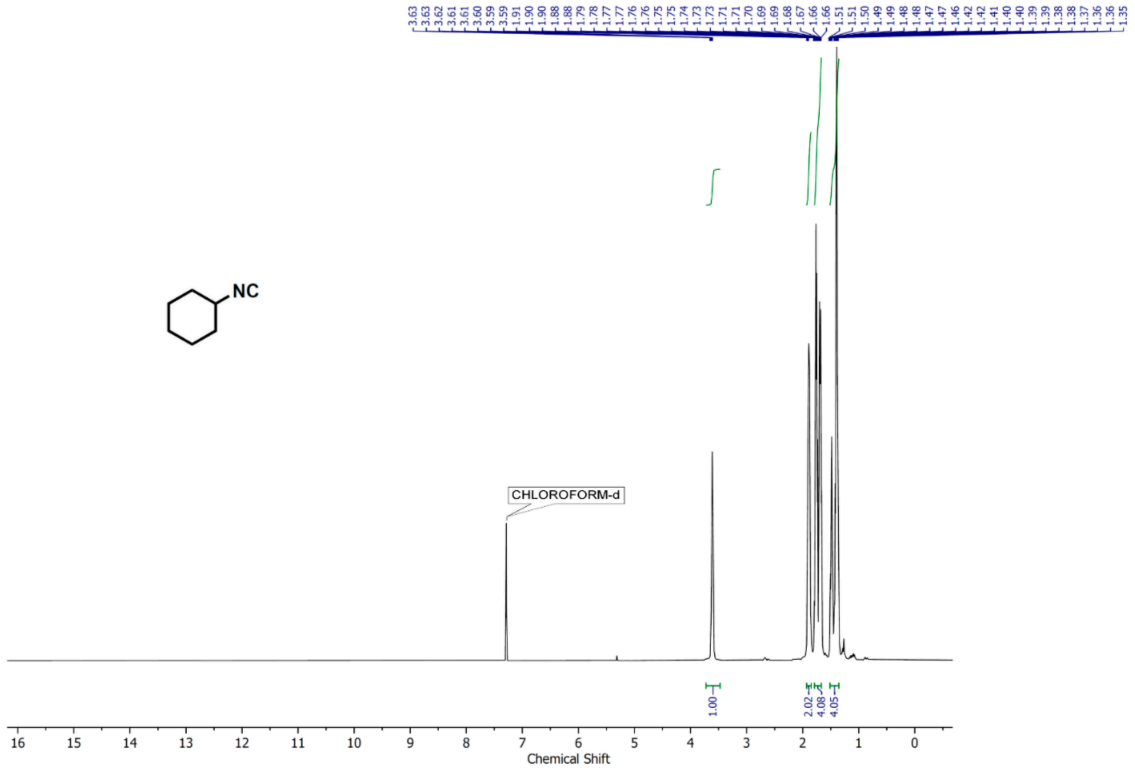

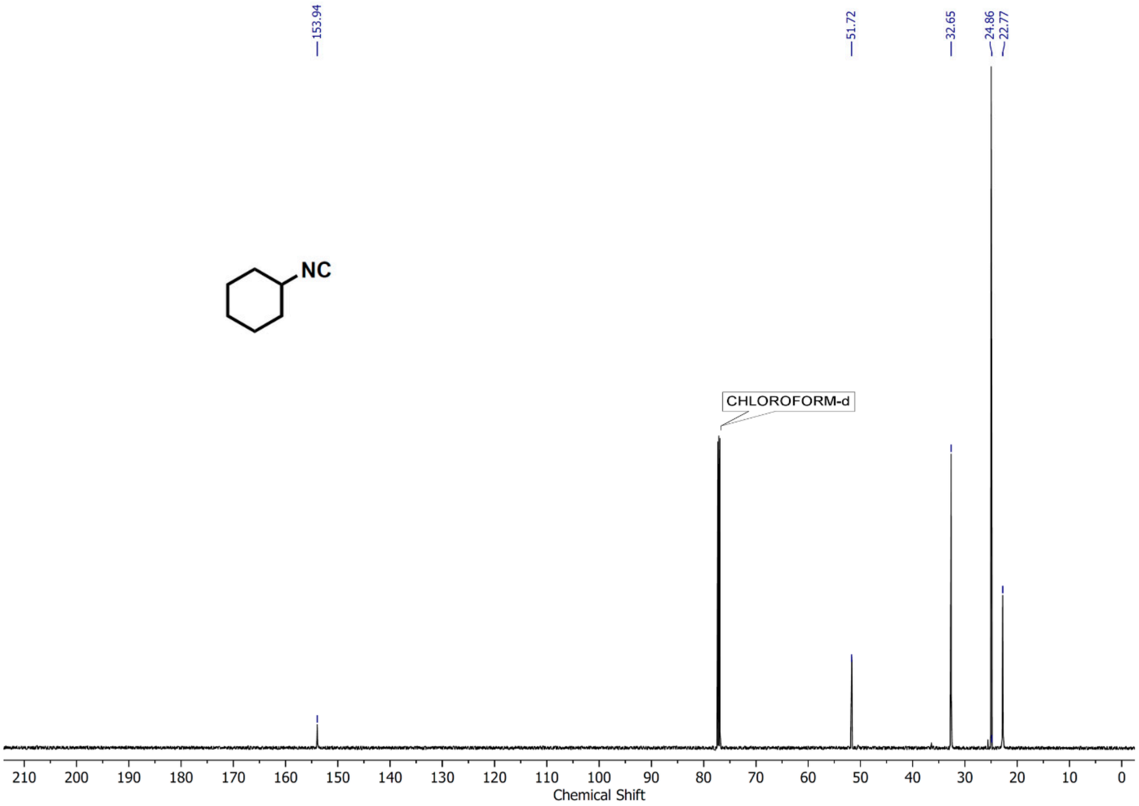

Figure S12. ^{1}H NMR (600 MHz, $CDCl_3$) and ^{13}C NMR (151 MHz, $CDCl_3$) of compound I-12

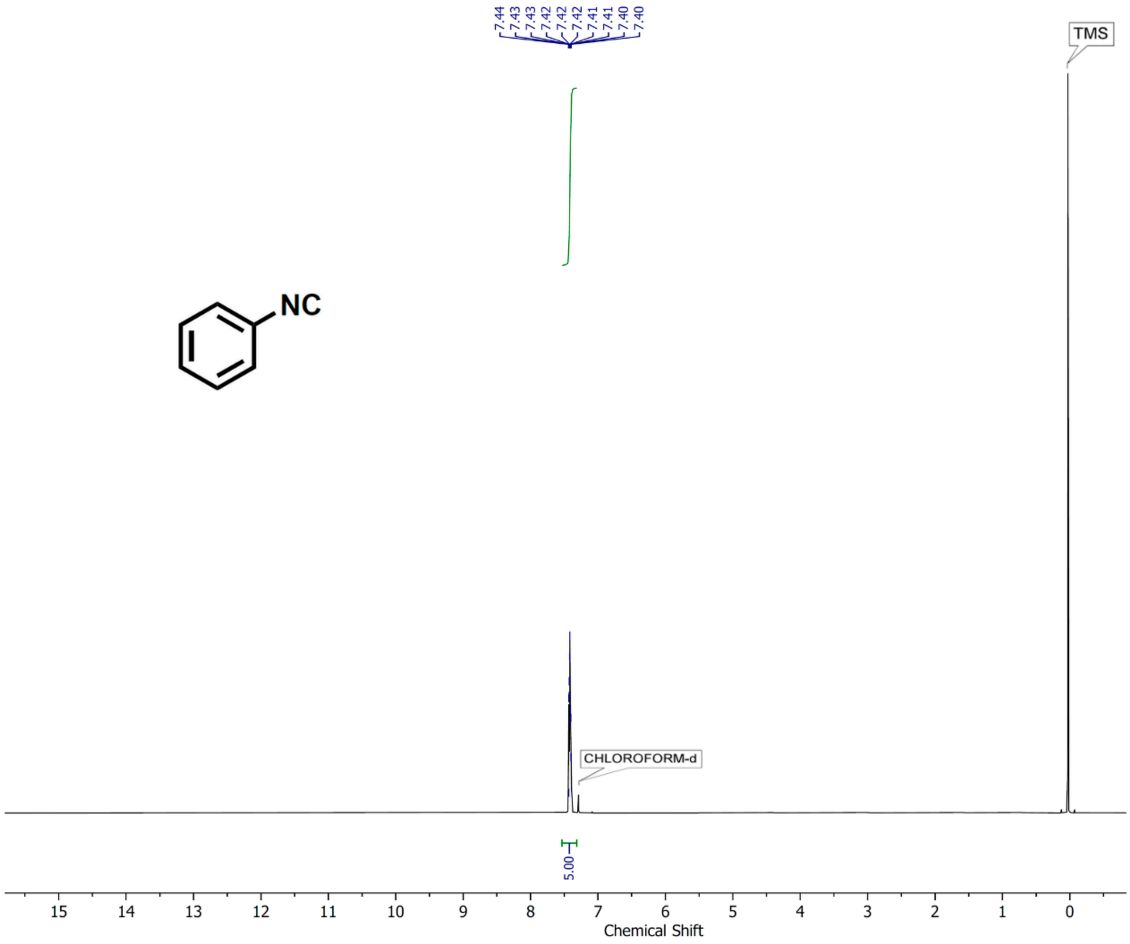

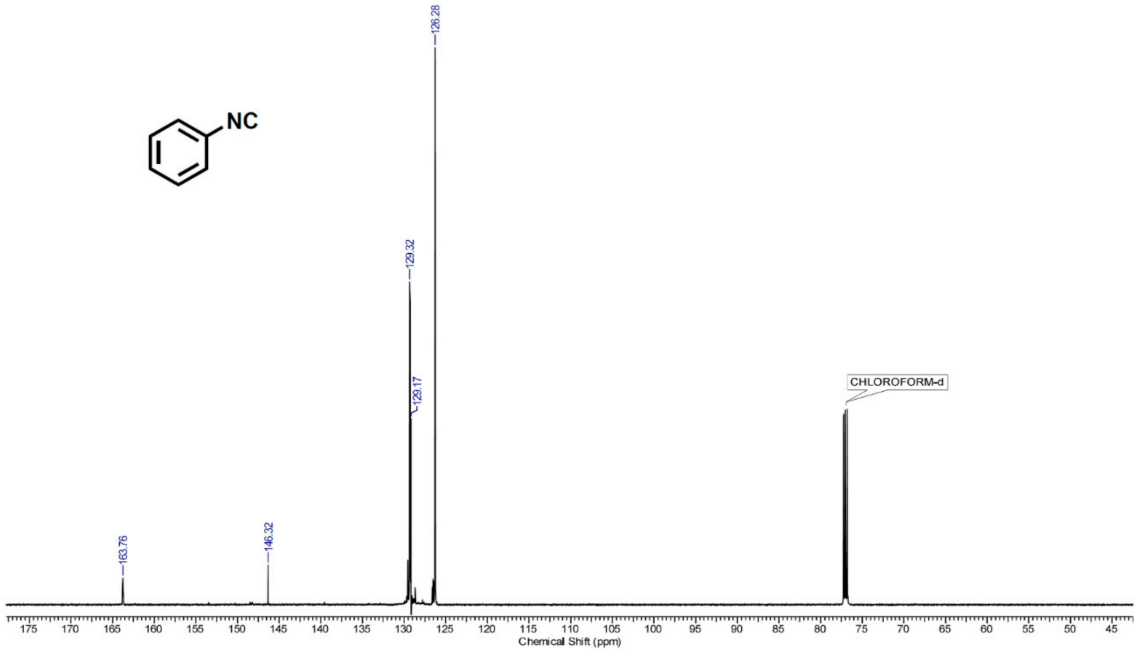

Figure S13. ^{1}H NMR (600 MHz, $CDCl_3$) and ^{13}C NMR (151 MHz, $CDCl_3$) of compound I-13

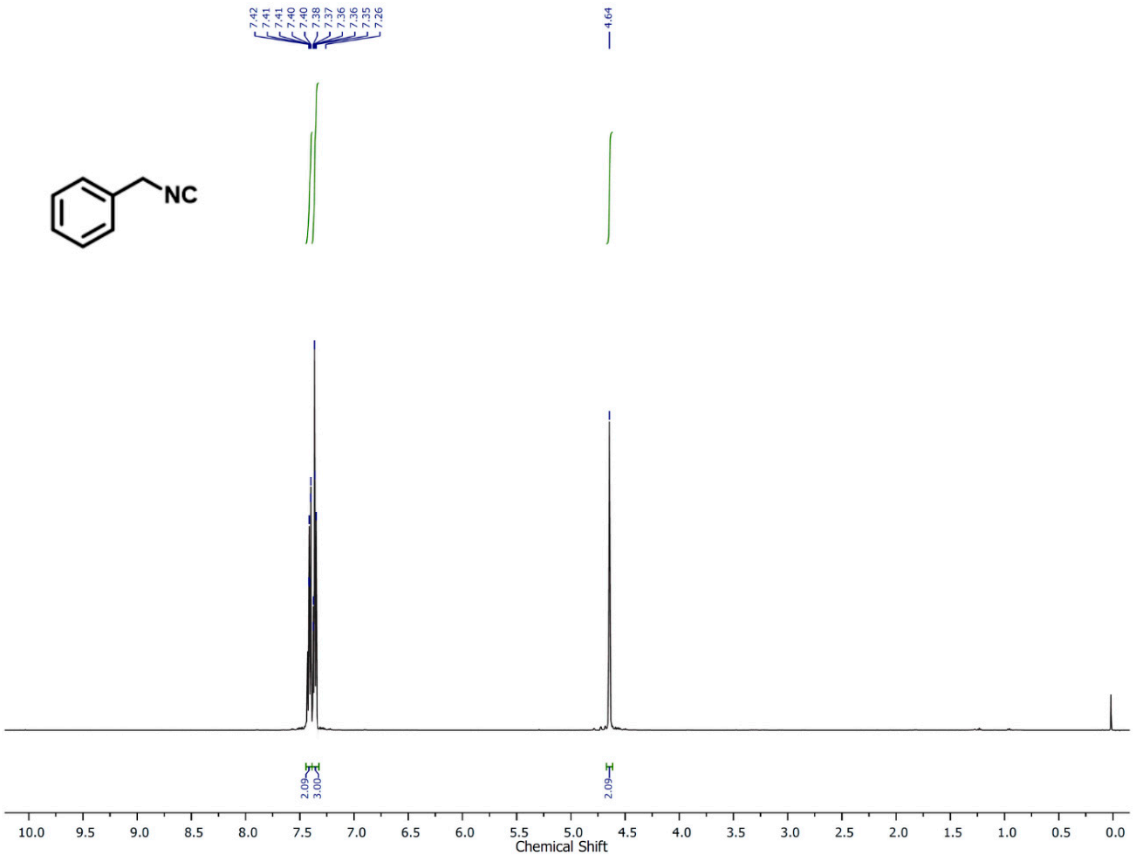

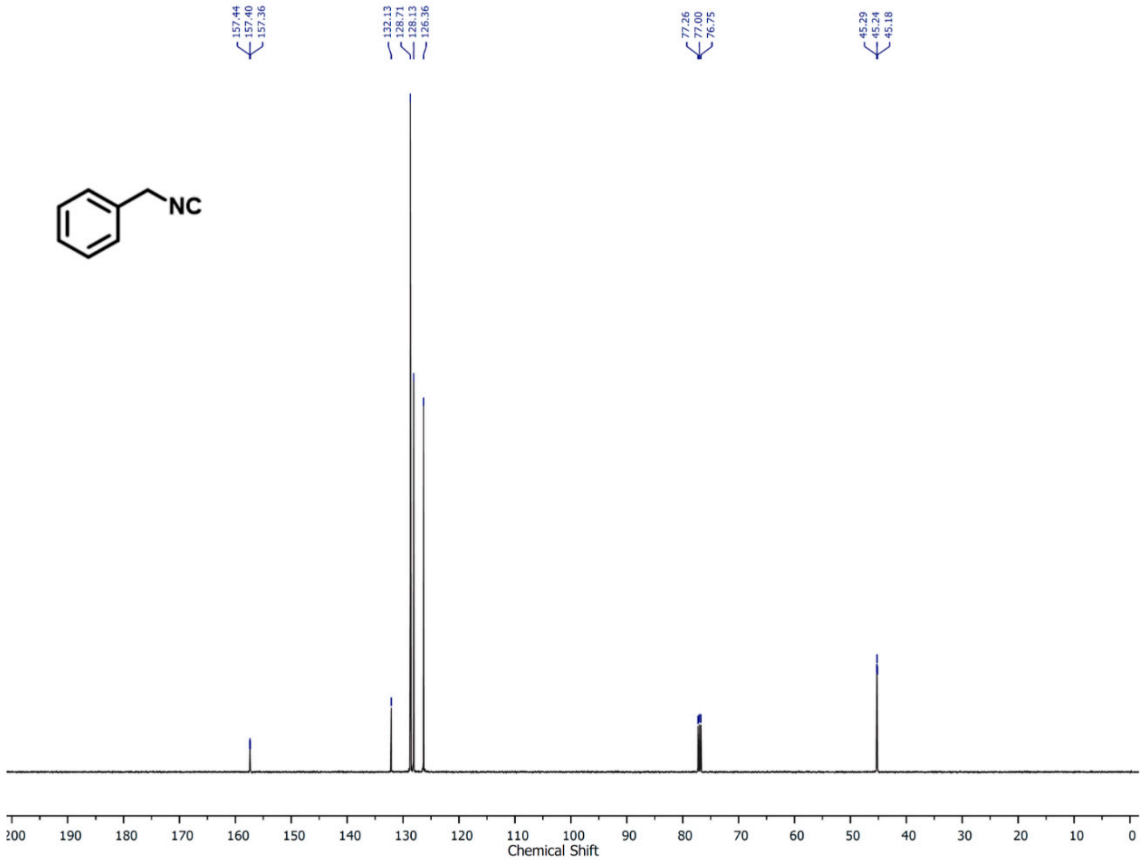

Figure S14. ^{1}H NMR (600 MHz, $CDCl_3$) and ^{13}C NMR (151 MHz, $CDCl_3$) of compound I-14

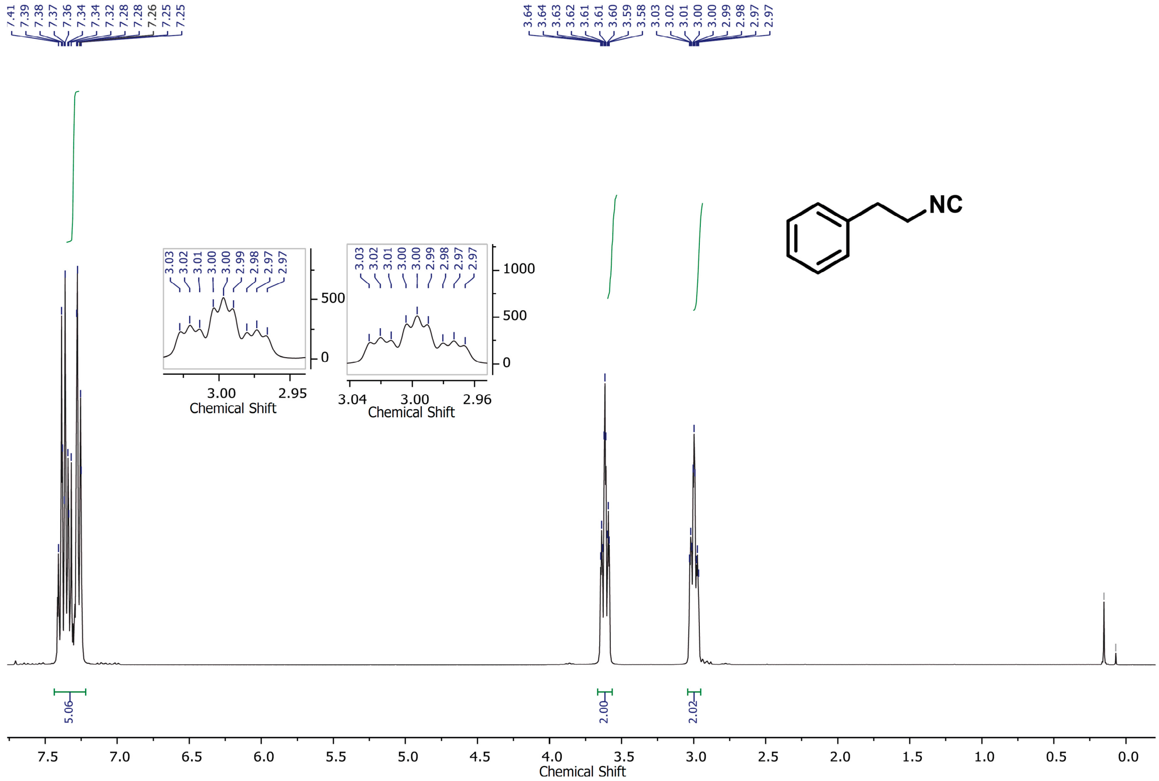

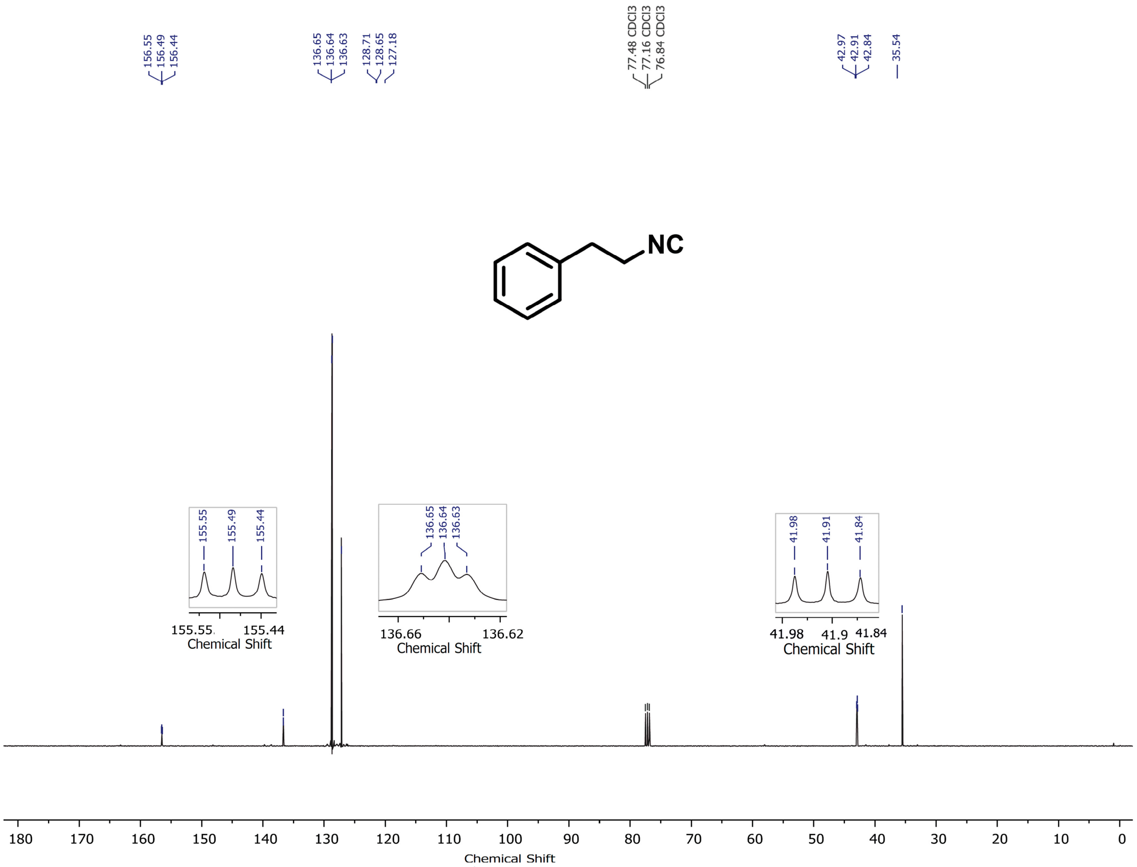

Figure S15. ^{1}H NMR (600 MHz, $CDCl_3$) and ^{13}C NMR (151 MHz, $CDCl_3$) of compound I-15

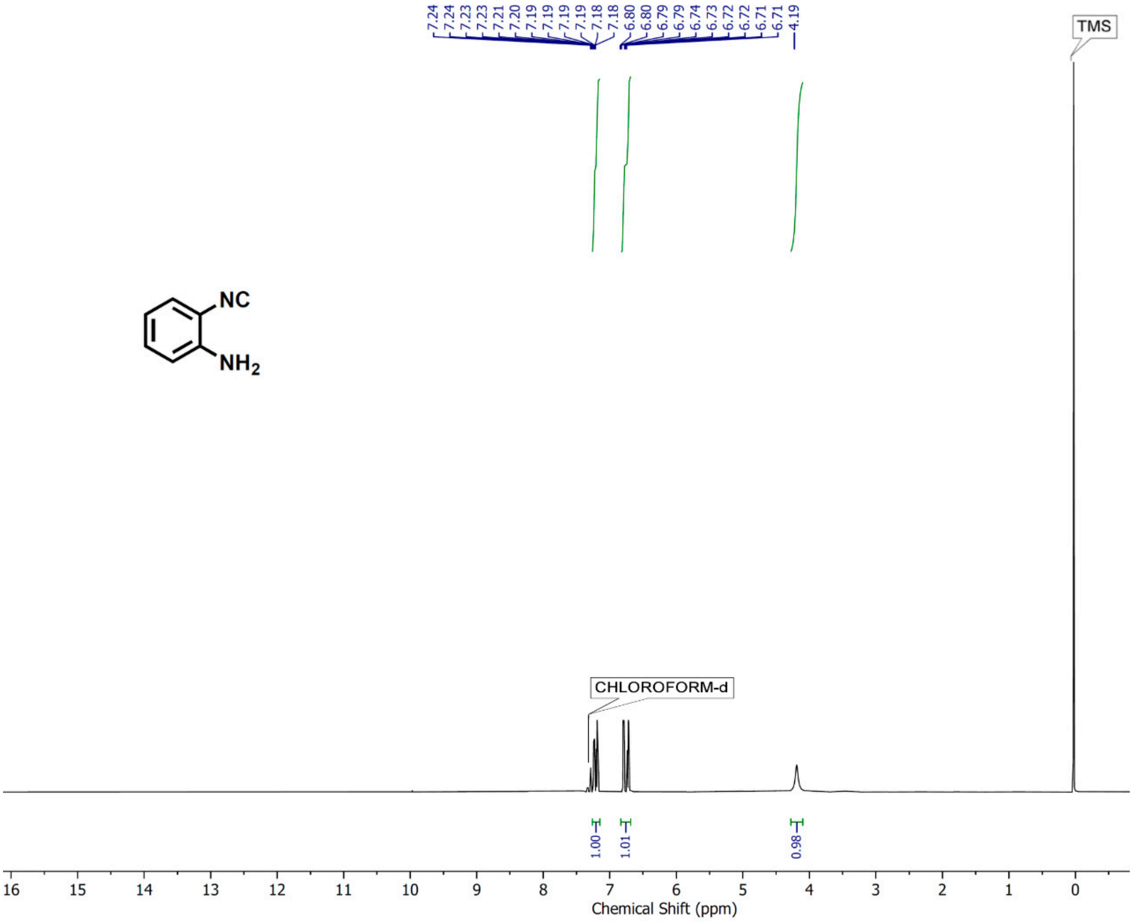

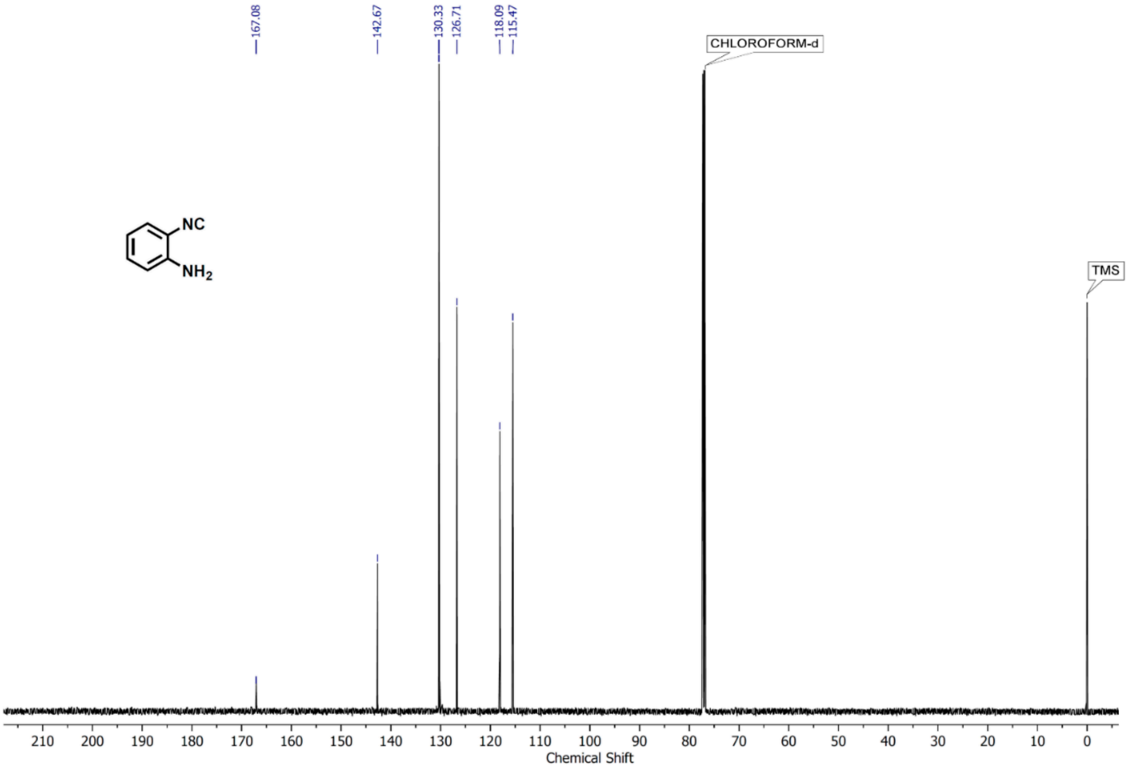

Figure S16. ^{1}H NMR (600 MHz, $CDCl_3$) and ^{13}C NMR (151 MHz, $CDCl_3$) of compound I-16

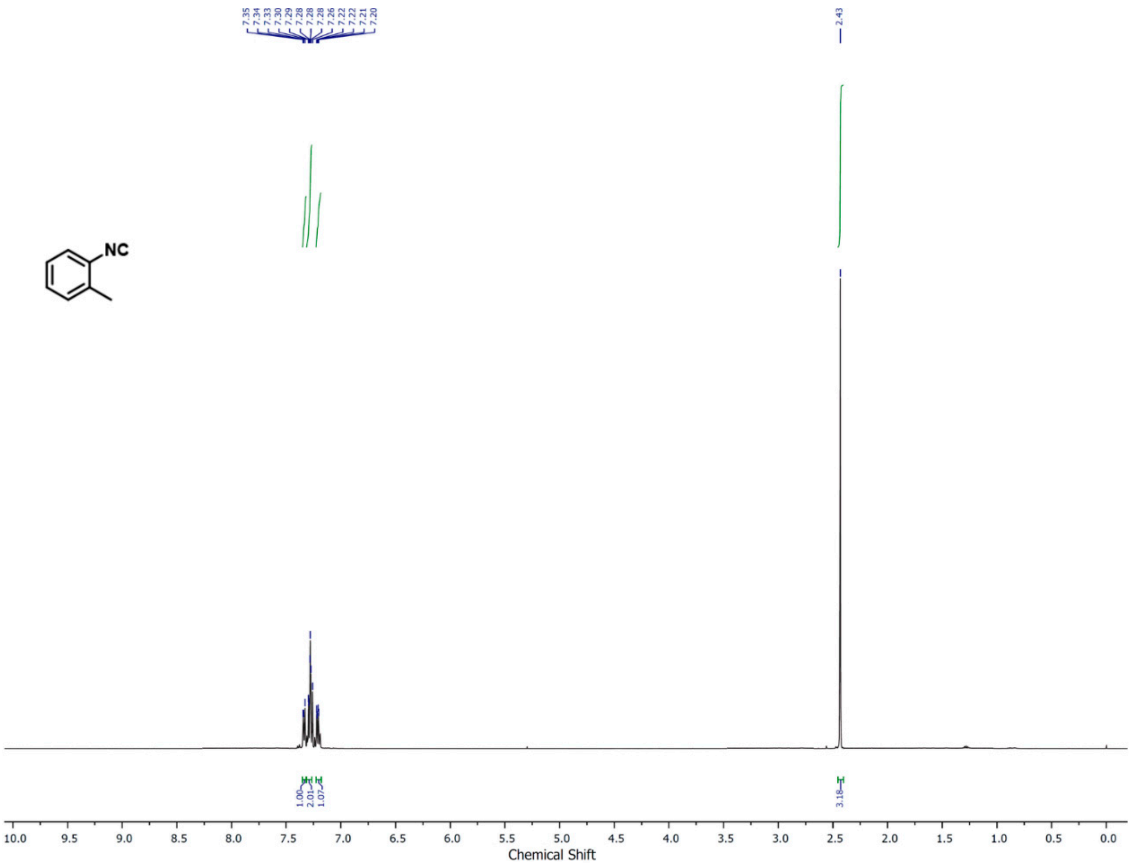

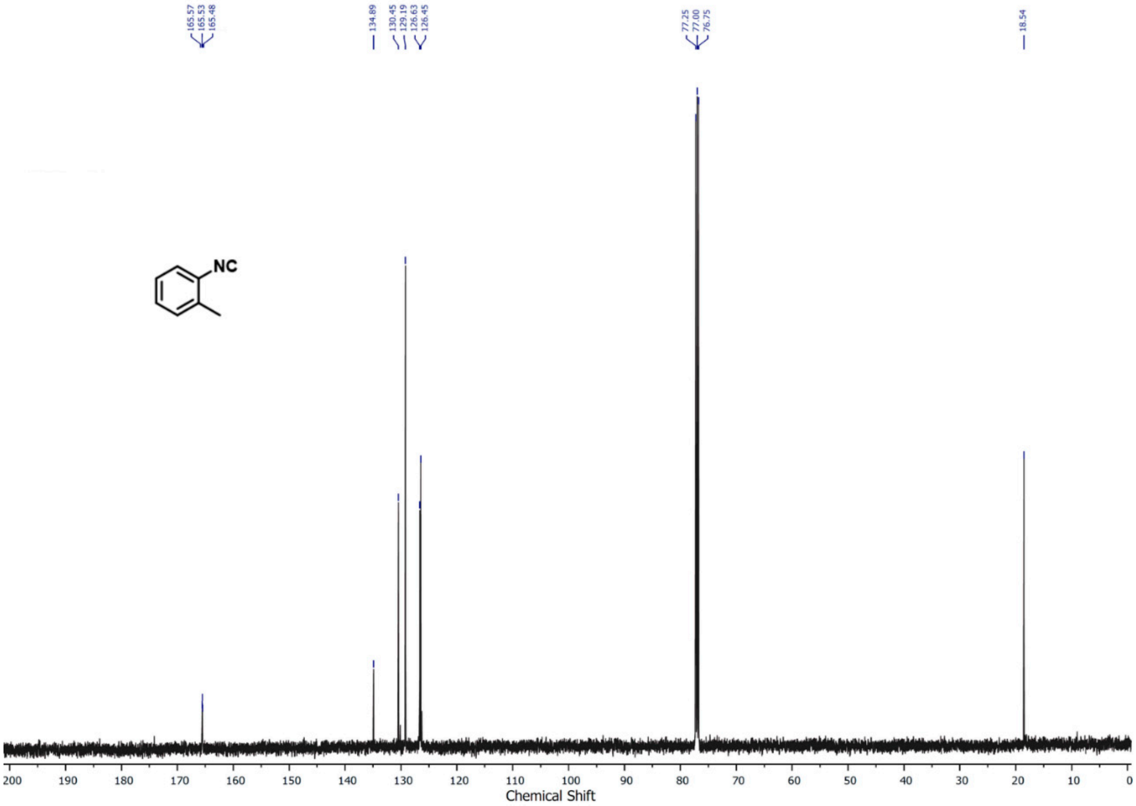

Figure S17. ^{1}H NMR (600 MHz, $CDCl_3$) and ^{13}C NMR (151 MHz, $CDCl_3$) of compound I-17

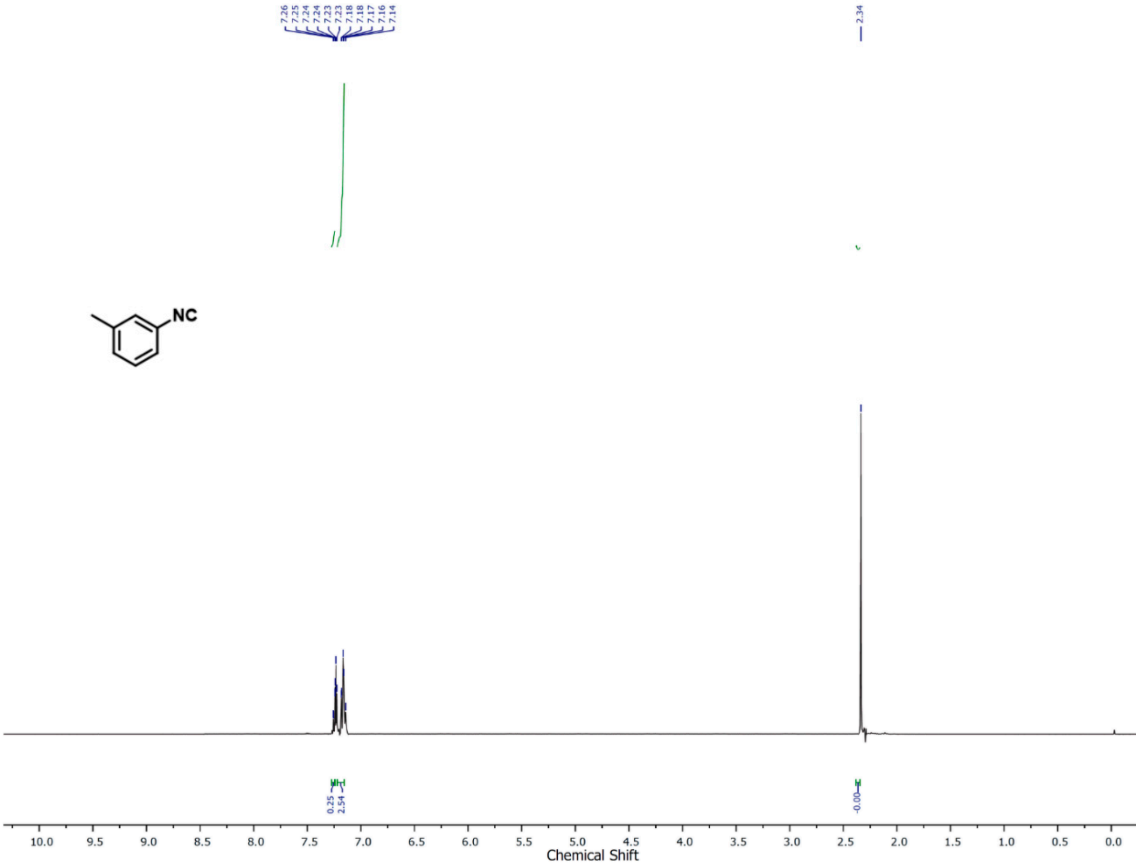

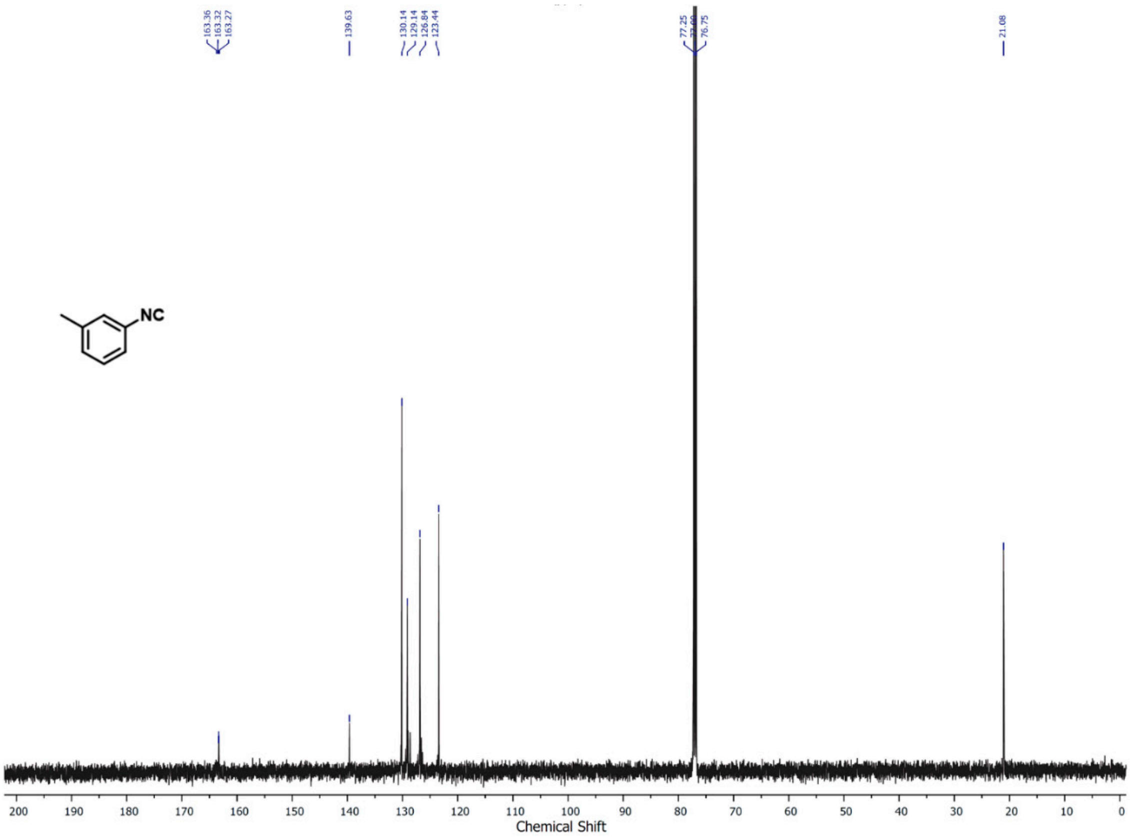

Figure S18. ^{1}H NMR (600 MHz, $CDCl_3$) and ^{13}C NMR (151 MHz, $CDCl_3$) of compound I-18

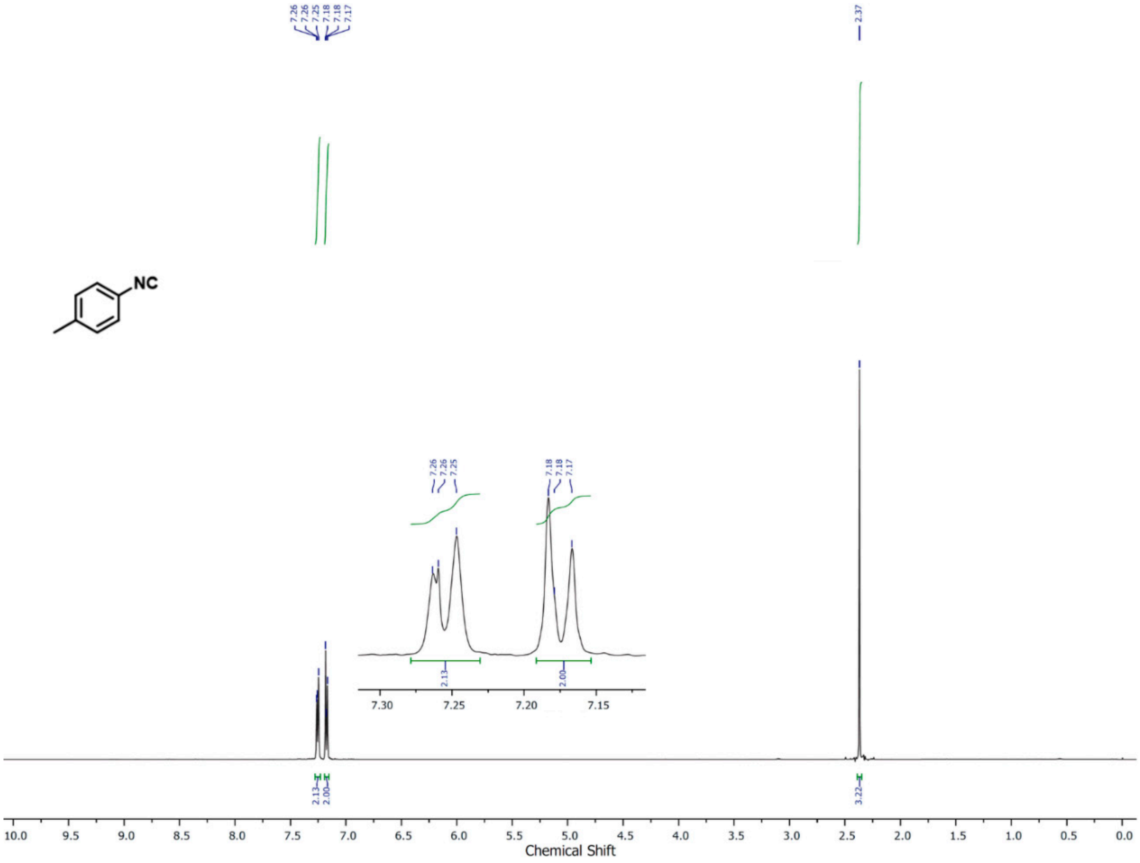

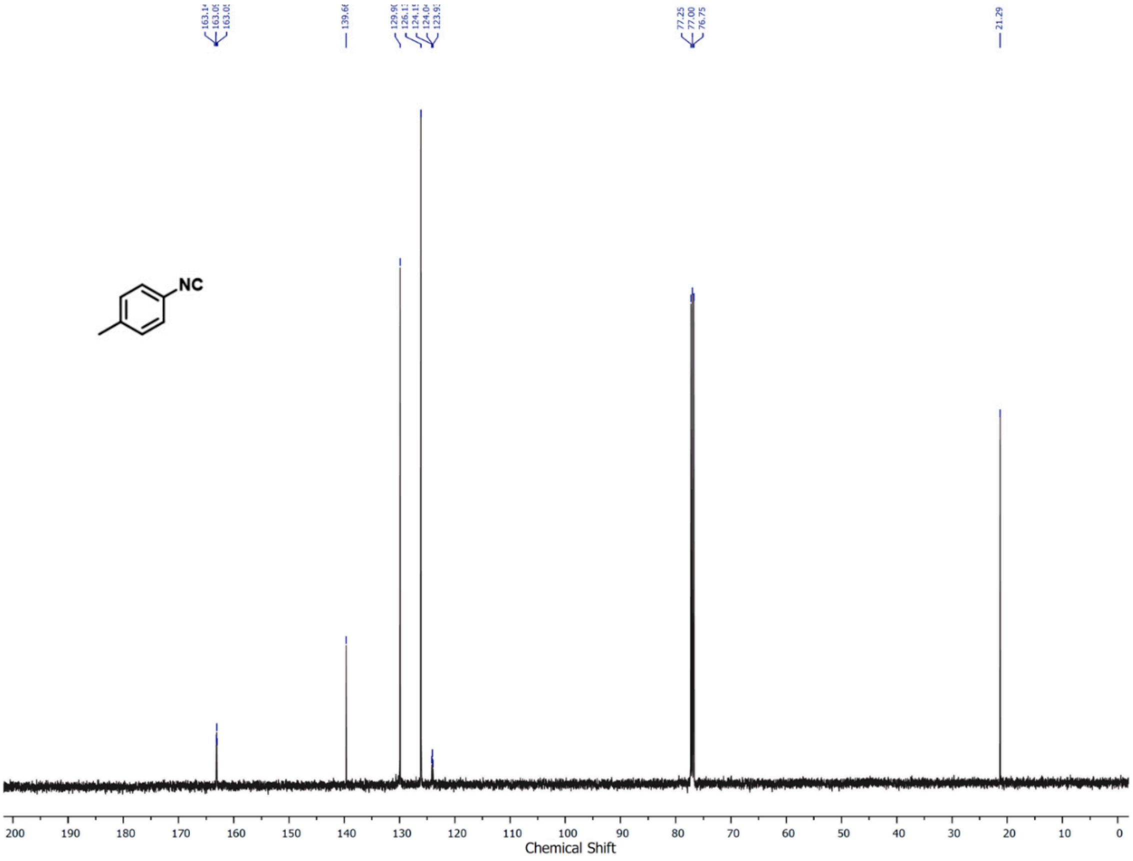

Figure S19. ^1H NMR (600 MHz, $CDCl_3$) and ^{13}C NMR (151 MHz, $CDCl_3$) of compound I-19

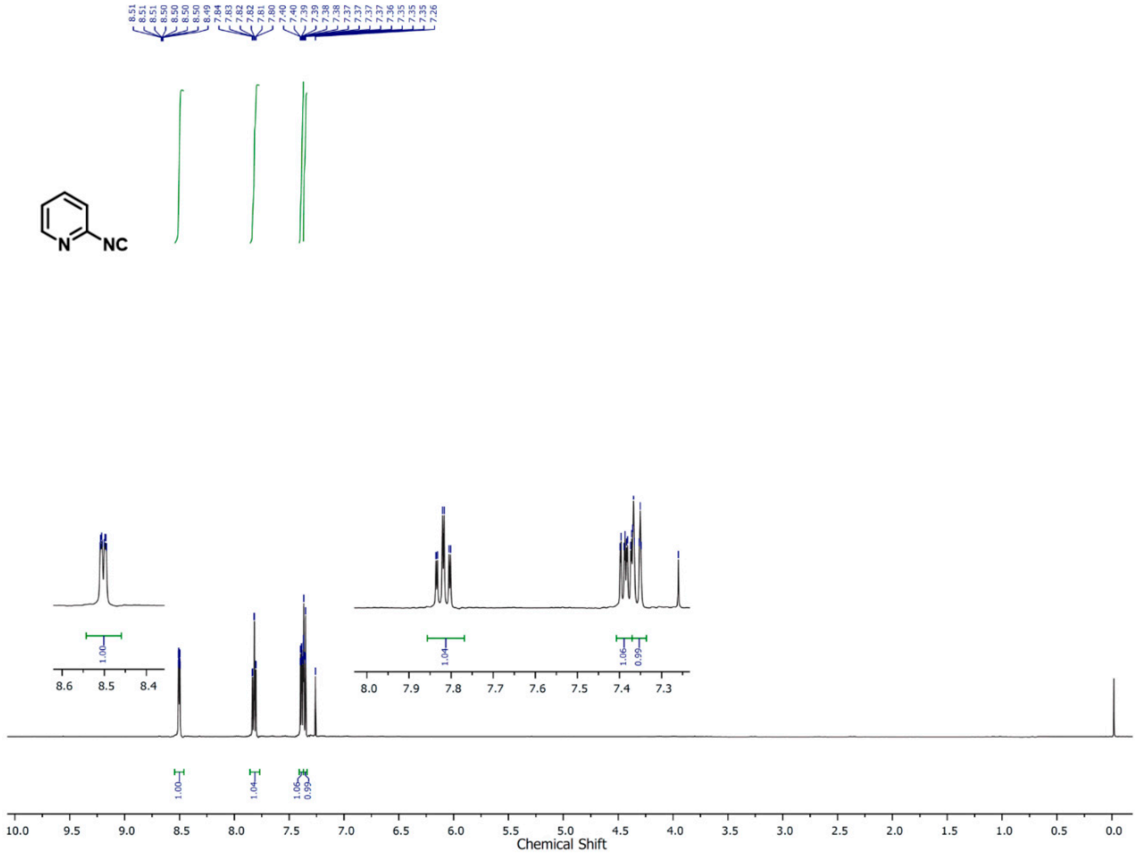

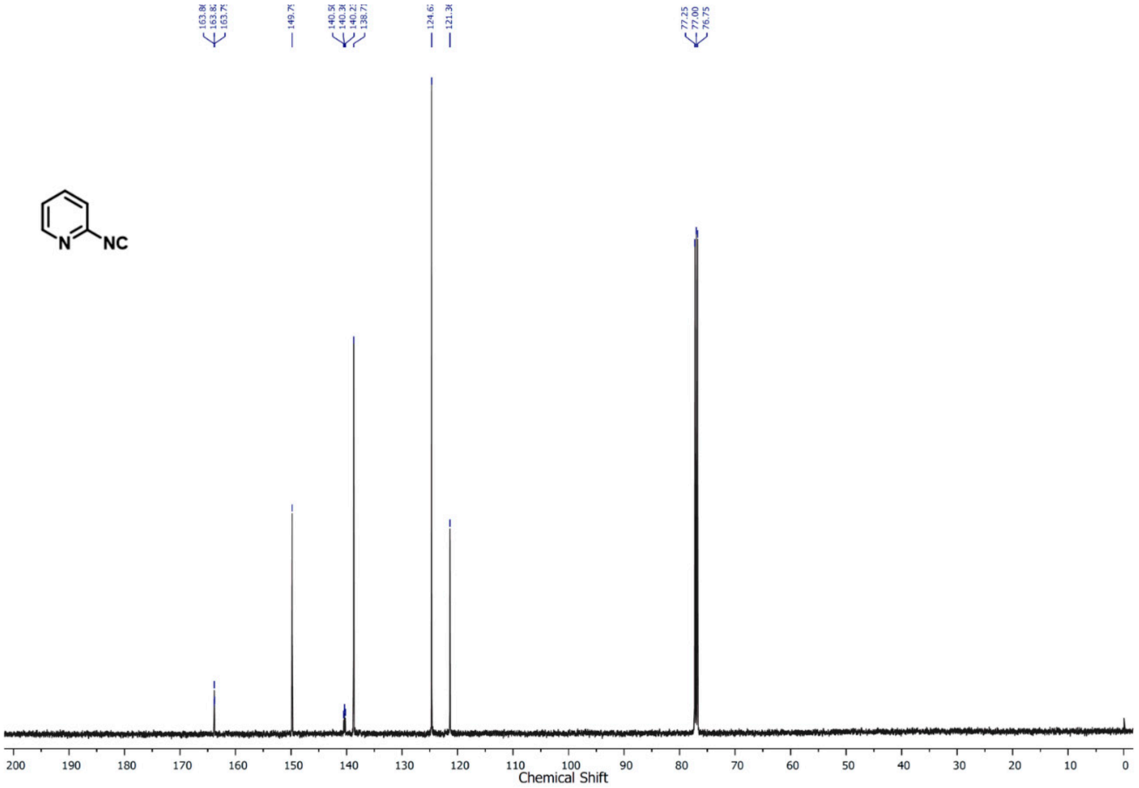

Figure S20. ^{1}H NMR (600 MHz, $CDCl_3$) and ^{13}C NMR (151 MHz, $CDCl_3$) of compound I-20

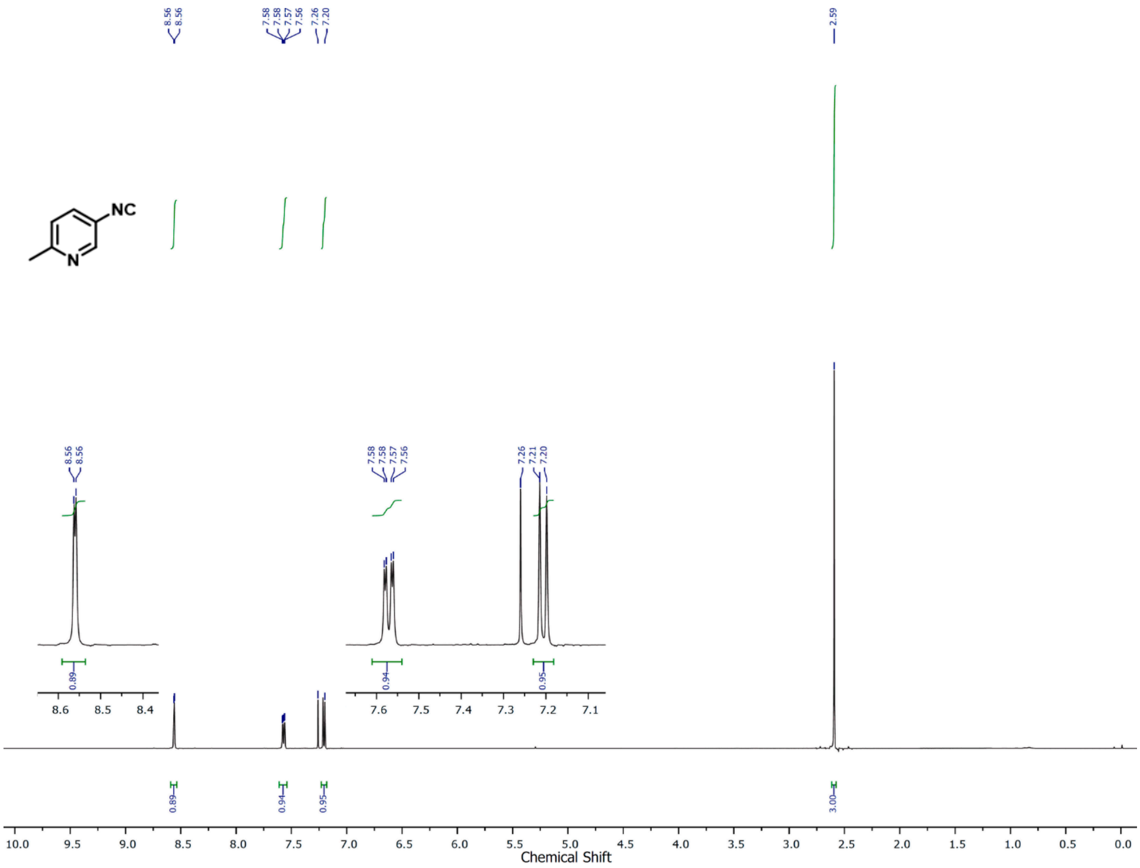

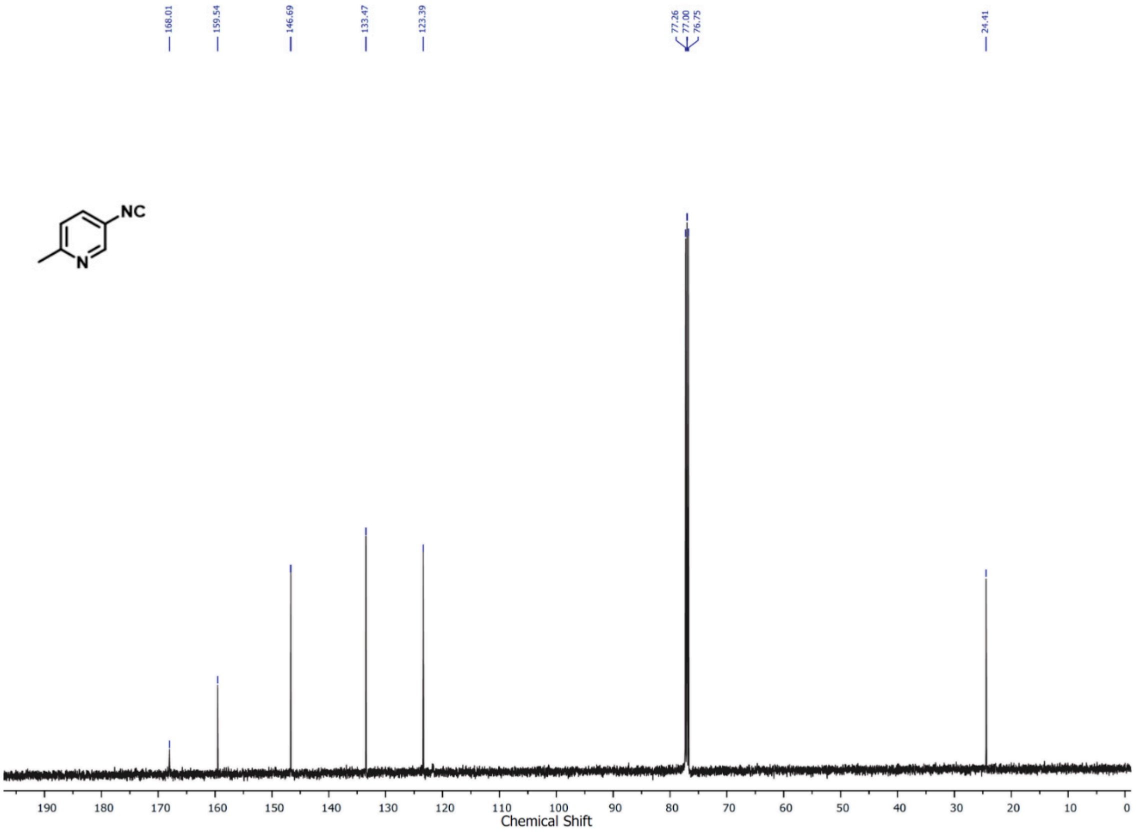

Figure S21. ^{1}H NMR (600 MHz, $CDCl_3$) and ^{13}C NMR (151 MHz, $CDCl_3$) of compound I-21
